# An Information–Theoretic Model of Abduction for Detecting Hallucinations in Explanations

**DOI:** 10.3390/e28020173

**Published:** 2026-02-02

**Authors:** Boris Galitsky

**Affiliations:** Knowledge Trail Inc., San Jose, CA 95127, USA; bgalitsky@hotmail.com

**Keywords:** hallucination detection, abductive reasoning, information theory, minimum description length, neuro-symbolic AI, discourse analysis, entropy-based inference

## Abstract

We present an Information–Theoretic Model of Abduction for Detecting Hallucinations in Generative Models, a neuro-symbolic framework that combines entropy-based inference with abductive reasoning to identify unsupported or contradictory content in large language model outputs. Our approach treats hallucination detection as a dual optimization problem: minimizing the information gain between source-conditioned and response-conditioned belief distributions, while simultaneously selecting the minimal abductive hypothesis capable of explaining discourse-salient claims. By incorporating discourse structure through RST-derived EDU weighting, the model distinguishes legitimate abductive elaborations from claims that cannot be justified under any computationally plausible hypothesis. Experimental evaluation across medical, factual QA, and multi-hop reasoning datasets demonstrates that the proposed method outperforms state-of-the-art neural and symbolic baselines in both accuracy and interpretability. Qualitative analysis further shows that the framework successfully exposes plausible-sounding but abductively unsupported model errors, including real hallucinations generated by GPT-5.1. Together, these results indicate that integrating Information–Theoretic divergence and abductive explanation provides a principled and effective foundation for robust hallucination detection in generative systems.

## 1. Introduction

Large Language Models (LLMs) have made substantial advances in natural language understanding and generation across diverse tasks. However, their practical use is limited by a persistent tendency to produce hallucinations—outputs that may be fluent and coherent yet factually incorrect or semantically implausible.

A broad range of techniques has been proposed for detecting unsupported or fabricated model outputs [1,2]. Existing methods are typically categorized as white-box, gray-box, or black-box. White-box approaches use internal representations or activation patterns to flag inconsistencies [3], but their dependence on model internals limits cross-model applicability. Gray-box approaches rely on intermediate signals such as token probabilities or entropy [4], though these signals often correlate imperfectly with factual correctness, especially in open-ended generation. Black-box methods, which examine only the generated text, are the most general but face their own limitations: external-knowledge approaches suffer from coverage gaps [5], and heuristic strategies such as self-consistency often fail when hallucinations are linguistically fluent and semantically coherent [6]. While many approaches to hallucination detection rely on external knowledge sources for fact-checking, several methods have been developed to operate in zero-resource settings, thereby eliminating dependence on retrieval. These methods rest on the premise that the genesis of LLM hallucinations is closely linked to the model’s intrinsic uncertainty. If one can estimate the uncertainty associated with the factual content produced by the model, hallucinations can often be detected without recourse to external evidence.

Uncertainty-based strategies generally fall into the following two categories:

1. LLM internal states. Internal model signals—such as token-level probabilities or entropy—serve as proxies for epistemic uncertainty [4]. Low-entropy generations tend to reflect confident, predictable continuations, whereas atypically high entropy may indicate unsupported or unstable content.
2. LLM behavioral variance. The studies elicit uncertainty behaviorally, either through natural-language self-assessment prompts [7] or through output-level variability. For example, Manakul et al. [8] detect hallucinations by sampling multiple responses to the same query and measuring the consistency of factual claims across samples.

**Figure 1:**
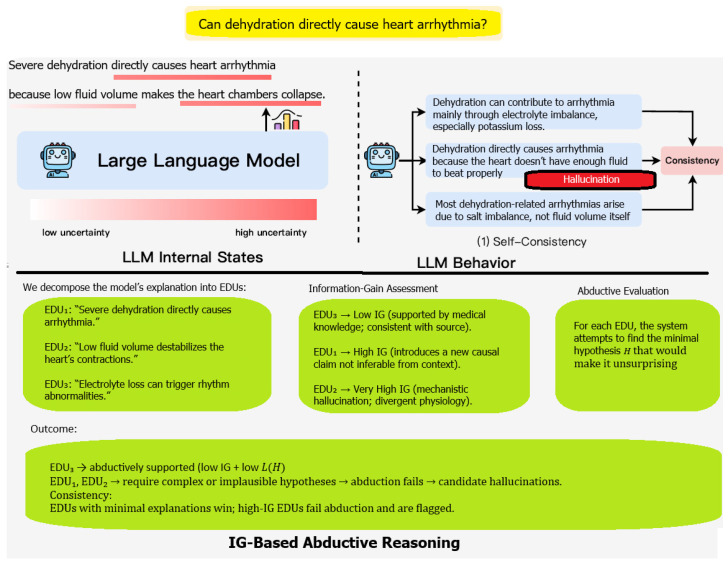
Illustration of our IG-based Abductive Reasoning approach (on the bottom) in comparison to LLM Internal States and LLM Behavior approaches. EDU—Elementary Discourse Units are used to assess the correctness of explanations.

Although such methods capture important uncertainty signals, they provide only local or surface-level indicators of instability. They do not explain why a claim is unsupported or what minimal hypothesis would be required for it to be true. This motivates our shift from merely estimating uncertainty to quantifying informational deviation and evaluating abductive plausibility. In particular, we extend uncertainty-based detection with an information-gain–driven abductive framework, where hallucinations are identified as claims whose informational divergence from the source cannot be justified by any computationally reasonable abductive hypothesis (Figure 1).

In this paper, we concentrate on a specific subclass of hallucinations that arise when a model produces claims that appear to be easily explainable by the given premises, even though the explanation is in fact incorrect. These are cases in which the model identifies a superficially plausible causal pathway connecting the premises to the conclusion, and—because the explanation is simple, salient, or heuristically attractive—treats it as valid. Crucially, the claim in question may still be factually true, yet the model’s justification for it is faulty. This makes the hallucination particularly insidious: it is not the claim’s truth-value that is compromised, but the inferential route by which the model arrives at it.

A paradigmatic example is the widely circulating misconception that walking in cold water can cause a gout attack. The model may generate the following reasoning: cold temperature → uric acid crystallization → gout flare. This explanation is coherent, compact, and causally intuitive—precisely the kind of abductive reasoning pattern that LLMs frequently overgenerate. However, the medical reality is substantially more complex: the combination of high temperature and low humidity had the greatest association compared with moderate temperature and average relative humidity [9]. Cold exposure alone does not precipitate gout; rather, gout flares arise from interactions among metabolic factors, urate load, local tissue dynamics, and inflammatory signaling. Cold may modulate symptoms indirectly, but it is not a straightforward causal trigger. Thus, while the conclusion (“I had a gout attack after walking in cold water”) could be true, the ease of the explanation masks its inaccuracy.

This phenomenon illustrates a central methodological challenge. Models tend to privilege explanations that are simple, available, and minimally costly from a cognitive perspective. When these low-complexity explanations align superficially with the structure of the premises, the model is likely to accept them uncritically—even when domain knowledge would rule them out. Our analysis, therefore, focuses on detecting hallucinations that stem not from fabricated facts, but from overly convenient abductive leaps: explanations that are too easy relative to the true causal structure underlying the domain.

**Figure 2:**
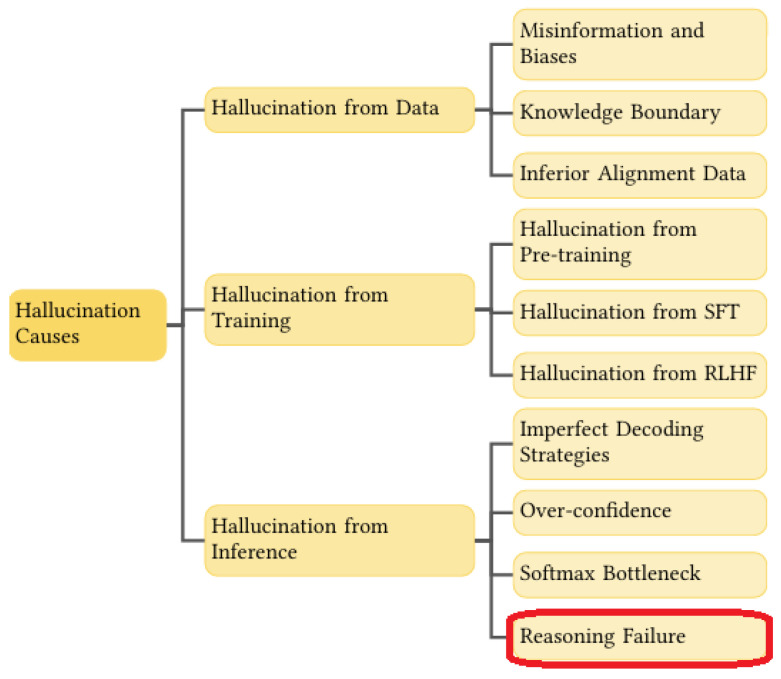
A taxonomy of hallucination types and the focus of this study. Red box indicates the focus of this paper.

The class of hallucinations we are tackling is shown in the bottom-right corner (Figure 2).

The effective use of knowledge in LLMs is fundamentally constrained by their underlying reasoning capabilities. In multi-hop question answering, for example, a model may possess all the relevant information yet still fail to produce accurate answers when multiple associative pathways exist between premises and the query [10]. Such ambiguity exposes the model’s limited ability to perform structured reasoning across intermediate steps. Moreover, (Berglund et al. [11]) identify a characteristic reasoning failure they term the Reversal Curse: while an LLM may successfully answer a query posed in the form “A is B,” it often fails to infer the logically equivalent converse, “B is A”. This asymmetry reveals a deeper deficiency that extends well beyond simple deductive transformations, indicating structural weaknesses in how LLMs represent and manipulate relational knowledge. This failure is even stronger in the case of “A explains B” and its reversal.

### 1.1. Example: Information-Gain Analysis of a Genealogical Hallucination

Although the focus of this paper is on explanation-based hallucinations, to illustrate how information gain identifies unsupported model inferences of plain facts, let us consider the query “Who are the grandmothers of Russian poet Alexander Pushkin?”. For this well-defined biographical question, authoritative sources converge on the following facts:

- Paternal grandmother: Olga Vasilyevna Chicherina;
- Maternal grandmother: Maria Alekseevna Pushkina (Hannibal).

However, YandexGPT produces the following hallucinatory response *H*_0_ (hallucinated output):

Alexander Sergeyevich Pushkin had two grandmothers—one on his father’s side and one on his mother’s side.

Maria Alekseyevna Ganibalkova (née Pushkina) (1745–1818)—paternal grandmother (*c*_1_), and Olga Vasilyevna Cherkasova (née Mineeva) (1737–1802)—maternal grandmother (*c*_2_).

Both names in *H*_0_ correspond to non-existent individuals, and neither appears in Pushkin scholarship or historical genealogical records. This discrepancy enables a clear demonstration of the information-gain framework.

Let S denote the distribution of plausible entities conditioned on verified biographical knowledge. An auditor model (e.g., NLI, QA, or a secondary LLM) assigns high probability mass to the true grandmothers (Chicherina; Pushkina/Hannibal) and effectively zero probability to fabricated entities such as Ganibalkova or Cherkasova–Mineeva. In contrast, the model’s response *R* = *H*_0_ commits strongly to these fabricated names, shifting nearly all probability mass toward non-existent individuals.

For the atomic claim “Pushkin’s paternal grandmother was Maria Alekseyevna Ganibalkova”, the auditor estimates:

*P*(Ganibalkova|*S*) ≈ 0 and *P*(Ganibalkova|*R*) ≈ 1.

The resulting information gain is, therefore, dominated by the following KL-divergence term:

*IG*(*c*_1_,*S*)=*DKL*(*P*(⋅|*R*)‖*P*(⋅|*S*)) ≈ log1/ϵ,

where ϵ is a small floor value used to avoid division by zero. In practice, this yields an *IG* score exceeding 13 bits, far above typical hallucination thresholds (1–5 bits). An analogous computation for the fabricated maternal grandmother (*c*_1_) yields a similarly high IG value. Aggregating claim-level scores—either by maximum or mean—produces a response-level information-gain estimate indicative of severe hallucination.

This example demonstrates the utility of IG-based detection: the model’s answer introduces entities that have no support in the source-conditioned distribution, resulting in extreme divergence between *P*(⋅|*S*) and *P*(⋅|*R*). Even without external databases, the probabilistic mismatch is sufficient to classify the response as hallucinated. The case thus provides a clear empirical instance of how information gain captures unsupported factual additions in generative model outputs.

### 1.2. Contribution

This work proposes a discourse-aware abductive reasoning framework for explanation-level hallucination detection, introducing a unified, formally grounded treatment of why an explanation should be accepted or rejected rather than merely whether a statement matches external facts. The key novelty lies in transforming explanation validation into a structured process of conditional justifiability, combining symbolic reasoning, discourse analysis, and probabilistic grounding. Whereas entropy-based methods are efficient in hallucination detection, this is the first study combining them with abduction and discourse analysis.

Our main contributions are as follows:

1. Explanation-level hallucination modeling via abduction. We formalize hallucination detection as an abductive reasoning problem: a model-generated explanation is accepted only if there exists a low-cost abductive hypothesis under which the explanation becomes entailed by the source context. This formulation goes beyond surface inconsistency detection by explicitly distinguishing legitimate hypothesis formation from genuine reasoning errors, a distinction that existing confidence- or consistency-based methods do not capture.
2. Discourse-weighted abductive scoring. We introduce a principled integration of Rhetorical Structure Theory (RST) into abductive reasoning. By assigning weights to Elementary Discourse Units (EDUs), the framework prioritizes nucleus-level claims and evidential relations while down-weighting peripheral or background material. These discourse weights modulate both information gain and abductive hypothesis cost, yielding explanations that are not only more factually precise but also more interpretable and aligned with human judgments of explanatory relevance.
3. Counter-abduction as logical defeasibility testing. We extend classical abduction with counter-abduction, explicitly generating rival hypotheses that compete to explain the same claim. An explanation is rejected when a counter-hypothesis achieves lower abductive cost, operationalizing the notion of defeasible reasoning. This adversarial mechanism provides a formal guarantee that accepted explanations are robust under evidential challenge, rather than merely self-consistent.
4. Probabilistic grounding via web-scale MDL estimation. We introduce a lightweight, distribution-free method for estimating abductive complexity using web-scale frequency statistics as a proxy for description length. Integrated into an MDL framework, this grounding mechanism generalizes traditional fact checking into an open-domain probabilistic confirmation signal, enabling explanation verification without reliance on curated knowledge bases or domain-specific retraining.

Collectively, these contributions establish a computationally grounded framework for hallucination-resistant explanation verification, applicable across high-stakes domains such as medicine, law, and scientific reasoning. By explicitly modeling explanatory plausibility, discourse importance, and defeasible alternatives, the proposed approach advances beyond post hoc fact checking toward human-aligned reasoning validation.

### 1.3. Comparison to Related Approaches

Unlike IG-only hallucination detectors, which rely solely on information–theoretic surprise or uncertainty signals [12,13], our framework does not equate high information gain with incorrectness. IG-only methods effectively detect unsupported additions but systematically fail in cases where a claim is surprising yet legitimately explainable through implicit background knowledge. By coupling IG with abductive hypothesis search, our approach distinguishes surprise that admits a simple explanation from surprise that reflects genuine reasoning failure.

Conversely, abduction-only verifiers [14,15] focus on logical entailment under hypothesized assumptions but lack a principled mechanism for penalizing overly convenient or ad hoc explanations. As a result, they tend to accept explanations that are logically repairable but epistemically implausible. Our integration of information gain, discourse weighting, and MDL-based hypothesis cost directly addresses this limitation by rejecting explanations that require structurally insufficient or narratively “too easy” abductive repairs.

In contrast to both lines of work, the proposed framework combines surprise, explanatory cost, discourse centrality, and defeasibility (via counter-abduction) into a single scoring and validation process. This joint treatment enables reliable detection of easy-but-wrong explanations—a failure mode that neither IG-only nor abduction-only approaches are designed to capture in isolation.

## 2. Information–Theoretic Formalization of Abduction

Abductive inference is traditionally understood as a qualitative process in which a reasoner selects the most plausible explanation for an observed fact. Classical philosophical treatments—from Peirce’s early writings to contemporary accounts of Inference to the Best Explanation (IBE)—identify several normative criteria for evaluating candidate explanations, including *simplicity*, *coherence*, *plausibility*, and *explanatory power* (Peirce 1878; 1903) [16,17]. While these guidelines capture the intuitions behind abductive reasoning, they lack precise quantitative definitions and therefore resist operationalization in computational systems. Recent work has demonstrated that information theory provides a principled mathematical foundation capable of formalizing these criteria and turning abduction into an optimization problem over measurable quantities.

Information theory treats inference as a process of minimizing uncertainty and encoding data as efficiently as possible. Within this view, hypotheses are evaluated based on how effectively they compress the information contained in observations. This perspective naturally aligns with the key abductive desiderata. First, simplicity corresponds to the *description length* of a hypothesis: shorter, less complex hypotheses carry a lower bit-cost and are therefore preferred according to the MDL principle. Second, explanatory adequacy is reflected in the *conditional entropy* of the observation given the hypothesis, *H*(O|H); a hypothesis that predicts or entails the observation well leaves little residual uncertainty and thus has low conditional entropy. Third, coherence (the degree to which the hypothesis and observation mutually support one another) maps onto *mutual information*, *I*(*H*;*O*), which quantifies how much knowing one reduces uncertainty about the other.

The plausibility of a hypothesis is naturally encoded as its *prior probability* within a probabilistic framework; plausible hypotheses have low information content (high prior, low −log *P*(*H*) and therefore contribute minimally to the total encoding cost. Finally, surprise reduction, a central feature of explanatory reasoning, corresponds to maximizing likelihood or minimizing the negative log-likelihood of the data, thus reducing the number of bits required to encode surprising events. Together, these correspondences establish a direct mapping between abductive criteria and information–theoretic quantities (Table 1).

By grounding abductive reasoning in measurable information–theoretic terms, we can formalize the selection of “best explanations” as a minimization of total encoding cost or, equivalently, as an optimization over uncertainty reduction. This yields computationally tractable objectives, such as MDL-based scoring or mutual-information–based selection, that directly instantiate the philosophical criteria of abduction. The result is a rigorous, unified account in which explanatory goodness is quantified through entropy, likelihood, and description length—allowing abductive inference to be implemented, compared, and evaluated systematically across symbolic, probabilistic, and neuro-symbolic reasoning systems.

Let *O* be an observation and *H* a candidate explanatory hypothesis. Abduction chooses

H* = arg max*_H_* Expl(*H*,*O*).

Information theory allows us to turn “explanatory quality” into a measurable objective.

The MDL principle states:

H* = arg min*_H_* [*L*(*H*) + *L*(*O*|*H*)],

where

- *L*(*H*) is the number of bits needed to encode the hypothesis;
- *L*(*O*|*H*) is the number of bits needed to encode the data given the hypothesis.

Abduction becomes choosing the hypothesis that yields maximum compression. Equivalently:

*L*(*O*|*H*) = −log *P*(*O*|*H*)

Thus, abduction maximizes the likelihood with a model complexity penalty.

We now express entropy-based explanation quality. The entropy of observation is expressed as

*H*(*O*) = −∑*xP*(*x*) log*P*(*x*)

Conditional entropy under a hypothesis:

*H*(*O*|*H*) = −∑*xP*(*x*|*H*) log*P*(*x*|*H*)

A good explanation minimizes conditional entropy:

H* = arg min*_H_ H*(*O*|*H*)

Equivalently, this H* hypothesis makes the observation least surprising.

Also, mutual information measures the explanatory power:

*I*(*H*;*O*) = *H*(*O*) − *H*(*O*|*H*)

Thus:

H* = arg max*_H_ I*(*H*|*O*)

The best explanation is the one that provides the largest entropy reduction.

### Bayesian Surprise and Abductive Shift

Bayesian surprise [18] can be expressed as

*S* = *D_KL_*(*P*(*H*|*O*) ‖ *P*(*H*))

An abductive hypothesis should induce a high posterior shift, but with a low description-length cost. Hence, the combined objective:

H* = arg max*_H_ I*(*H*;*O*) − *L*(*H*)

**Figure 3:**
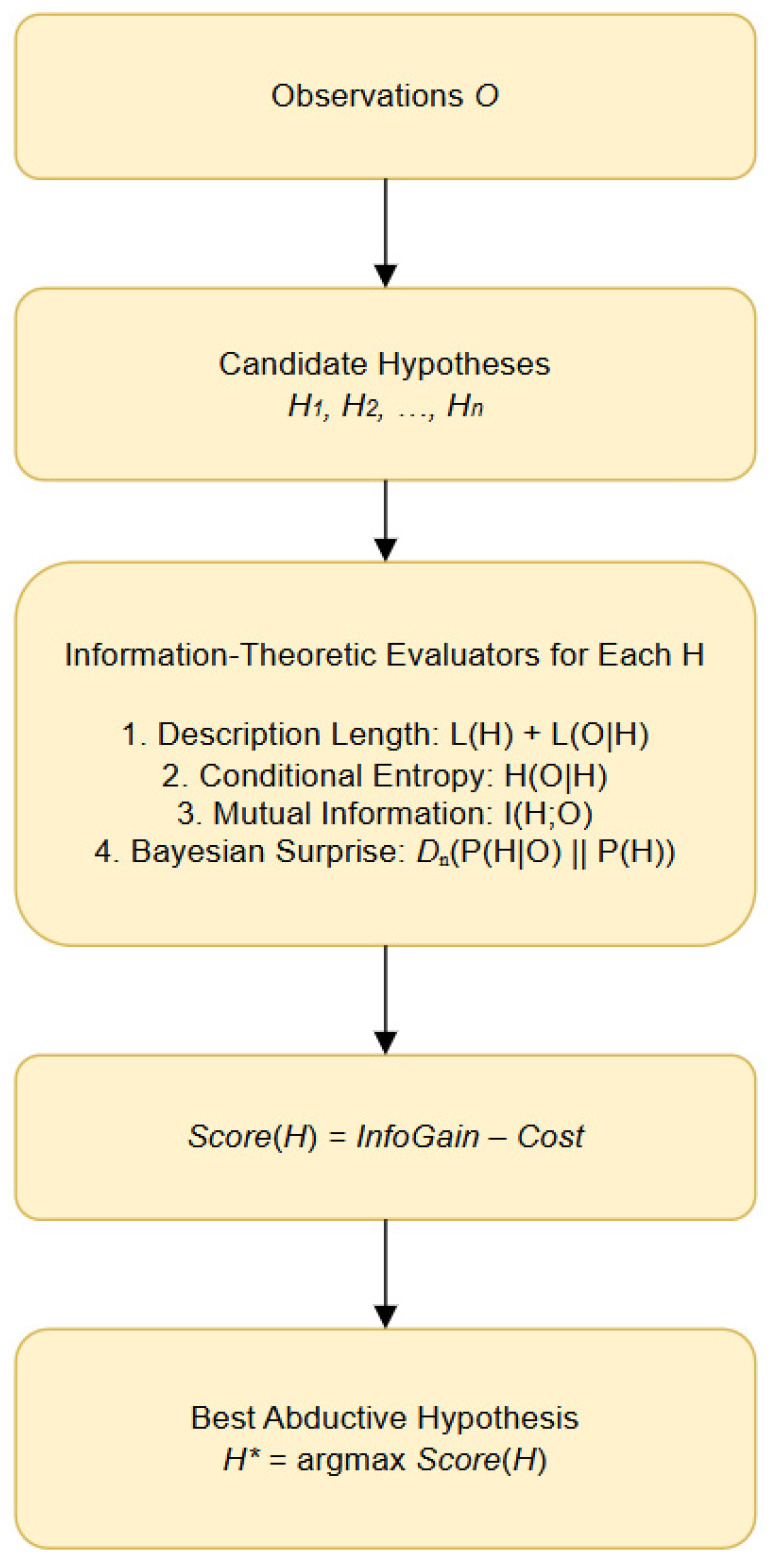
An algorithm for abduction + information–theoretic formalization (see Appendix A for more details).

This expression unifies informativeness, simplicity, and explanatory adequacy, providing a fully information–theoretic formalization of abduction (Figure 3).

## 3. Abduction as a Structural Corrective Layer for Chain-of-Thought Reasoning

Chain-of-Thought (CoT) prompting has become a dominant strategy for eliciting multi-step reasoning from LLMs. By encouraging models to articulate intermediate steps, CoT aims to expose the latent reasoning trajectory behind a prediction. However, numerous empirical analyses suggest that CoT outputs often reflect post hoc *narratives* rather than veridical reasoning traces. Because CoT unfolds autoregressively, each step is strongly influenced by the preceding linguistic surface form rather than by an internal, constraint-driven reasoning structure. This generates characteristic failure modes: invented premises, circular justifications, incoherent jumps between steps, and a high degree of variance under paraphrase. As a result, CoT explanations may be fluent and plausible but lack global coherence or factual grounding.

Abductive reasoning provides a natural remedy for these limitations because it is explicitly designed to construct the *best available explanation* for a set of observations under incomplete information. Unlike deduction, which propagates truth forward from known rules, or induction, which generalizes from samples, abduction seeks hypotheses that make an observation set minimally surprising. When integrated with LLMs, abduction can serve as a structural corrective layer that aligns free-form CoT text with formal explanatory constraints. The goal is not merely to post-verify LLM output but to reshape the generative trajectory itself, yielding reasoning paths that are coherent, defeasible, and governed by explicit rules.

In a neuro-symbolic pipeline, the role of abduction is to constrain the model’s reasoning space, reveal implicit assumptions, and ensure that the chain as a whole satisfies the explanatory minimality principles characteristic of abductive logic programming and related frameworks (e.g., probabilistic logic programming, argumentation-based abduction, and paraconsistent abduction). The resulting system treats CoT not as a static artifact but as a dynamic structure subject to revision, hypothesis insertion, and consistency checking. This greatly mitigates classical CoT hallucinations, particularly those involving unjustified intermediate premises.

LLMs exhibit several well-documented weaknesses in generating extended reasoning chains:

1. Local coherence without global consistency. Autoregressive generation ensures that each step is locally plausible, but the chain as a whole often lacks a unifying explanatory structure. This makes even long chains susceptible to hidden contradictions.
2. Narrative drift. The model may start with a plausible explanation but gradually drifts toward irrelevant or speculative content, especially when confronted with ambiguous or incomplete premises.
3. Invented premises and implicit leaps. Because LLMs are rewarded for fluent continuations, they may introduce explanatory elements that have no grounding in the problem context.
4. Inability to retract or revise past steps. CoT is monotonic: once a step is generated, the model rarely revises it when new evidence appears.
5. Lack of minimality. CoT chains often include redundant or extraneous content that weakens verifiability and expands the space for hallucination.

These deficiencies reflect the absence of a symbolic structure guiding the explanation. They are symptoms of the “language-model fallacy”: the assumption that linguistic plausibility implies logical validity. Abduction directly targets these pathologies.

### Abduction as a Missing-Premise Engine

One of the most powerful contributions of abduction to CoT reasoning is its ability to identify and supply *missing premises*. If the LLM asserts a conclusion for which no supporting evidence exists, the abductive engine detects the explanatory gap and suggests minimal hypothesis candidates to fill it. Because the goal in abduction is to construct the *best available* explanation rather than an arbitrary one, the resulting hypotheses must satisfy structural constraints: consistency with the domain theory, minimal additions, and coherence with all observations.

In practice, this mechanism serves two complementary purposes. First, it prevents the LLM from inventing arbitrary premises, because only hypotheses justified by the symbolic knowledge base are admissible. Second, it allows an LLM to maintain explanatory completeness even when the input is underspecified. Rather than hallucinating supporting details, the LLM can explicitly acknowledge abductive hypotheses, yielding transparent explanations that distinguish between observed facts and inferred assumptions.

This missing-premise correction is particularly valuable in domains such as medical reasoning, legal argumentation, or engineering diagnostics, where unjustified intermediate steps pose significant risks. The integration ensures that all steps in a CoT chain are grounded in either evidence or structured hypotheses. See Appendix B for more details.

## 4. Information Gain as a Framework for Hallucination Detection

Hallucinations in language model outputs typically arise when generated content introduces propositions that are not inferable from, or directly contradict, the source context. From an information–theoretic perspective, such responses exhibit disproportionately high *information gain* relative to the input: they contain informational content that is absent from the source and therefore cannot be epistemically justified. Intuitively, if a model produces statements that cannot, even in principle, be derived from the provided evidence, this “novel” information warrants suspicion and should be subjected to verification.

Formally, information gain (IG) is defined as the reduction in entropy of one distribution conditioned on another. For hallucination detection, we adapt this construct to quantify how much the model’s response *R* shifts a distribution of plausible world-states relative to that supported by the source *S*. Let *P*(⋅|*S*) denote the probability distribution over candidate factual states conditioned on the source, and *P*(⋅|*R*) the analogous distribution conditioned on the model’s response. The information gain introduced by the response is as follows:

*IG*(*R*,*S*) = *D_KL_*(*P*(⋅|*R*) ‖ *P*(⋅|*S*)) (1)

where *D_KL_* denotes the Kullback–Leibler divergence. High values of *IG*(*R*,*S*) signify that the response causes a substantial shift from the distribution justified by the source, thereby indicating the presence of unsupported or contradictory claims. In practical systems, these distributions are approximated using an “auditor,” such as an NLI model, a QA system, or a secondary LLM queried under controlled conditions.

Several implementation strategies can operationalize information–gain–based hallucination detection. A first approach uses a Natural Language Inference (NLI) model to evaluate the evidential status of atomic claims extracted from the response. After decomposing *R* into minimal propositions {*c*_1_, …, *c_n_*}, each claim is tested against the source. Claims that are entailed by the source correspond to low IG; those judged “neutral” represent unsupported additions with moderate to high IG; and contradictions yield very high IG, reflecting the strong divergence from source-conditioned expectations. Aggregating these scores across claims (e.g., by maximum or average IG over non-entailed claims) provides a robust, fine-grained hallucination signal.

A second strategy employs an LLM directly as a probability estimator. Here, approximate distributions *P*(⋅|*S*) and *P*(⋅|*R*) are constructed by prompting the auditor model with masked or scoring templates designed to elicit likelihoods over semantically salient tokens or propositions [19]. KL divergence between these distributions yields an IG estimate: large shifts imply that the response meaningfully alters the auditor’s posterior expectations beyond what the source supports.

A third, retrieval-augmented approach (El-Enen et al. [20]) reformulates hallucination detection as divergence between answers to structured queries. Queries are automatically derived from propositions in *R*. For each query *q_i_*, a QA model produces an answer based solely on the source (*A_s_*) and an answer based on the response (*A_r_*). The degree of mismatch between *A_s_* and *A_r_* serves as an IG proxy: equivalence indicates low IG (faithful), absence of a source-supported answer but a response-provided answer indicates high IG (unsupported), and direct conflict yields very high IG (contradiction).

This information–theoretic framing offers several advantages. It is grounded in a well-established theoretical construct—entropy reduction—and provides a principled explanation for why a given output should be deemed hallucinatory. It also affords fine-grained, claim-level attribution of error, making it suitable for applications requiring interpretability [21]. The method is model-agnostic and can be applied to the outputs of any generative system. Importantly, IG-based detection remains sensitive to subtle forms of hallucination that are factually correct in isolation but lack support from the given evidence.

However, several challenges must be acknowledged. The reliability of the approach is bounded by the accuracy of the auditor model: weak or hallucination-prone auditors can lead to erroneous IG estimates. The computational cost may be non-trivial, as many strategies require decomposition into atomic claims and multiple auditor queries. In open-ended dialogs, defining the source distribution *P*(⋅|*S*)) is non-trivial, particularly when the model legitimately leverages background knowledge. Finally, setting appropriate thresholds for IG remains task-dependent: excessively strict thresholds penalize legitimate abstraction and summarization, whereas lenient thresholds allow hallucinations to pass undetected.

Overall, the information gain framework reconceptualizes hallucination detection as a problem of measuring informational consistency between a source and a generated response. By quantifying how much the response departs from the evidence-supported probability distribution, this approach provides a theoretically grounded, explainable, and empirically effective mechanism for identifying unsupported model claims, especially in settings—such as summarization and retrieval-augmented generation—where faithfulness is central.

### 4.1. Abductive Reasoning with Entropy-Based Verification

While information gain provides a quantitative measure of how strongly a model’s response diverges from what is supported by the source, it does not by itself determine why the divergence arises or what explanatory commitments would be required for the response to be valid [22]. Abductive reasoning offers a complementary, logic-based mechanism for determining whether unsupported propositions can be justified through plausible explanatory hypotheses. Integrating entropy-based detection with abductive inference yields a unified neuro-symbolic framework in which hallucinations are characterized not merely by informational inconsistency but by the failure of minimal, coherent explanatory hypotheses to reconcile the response with the source.

Abduction—formalized as inference to the best explanation—selects hypotheses H that, if assumed, would render an observation O expectable. In the context of hallucination detection, the observation corresponds to an atomic claim extracted from the response, and the source context serves as the evidential baseline. A claim is deemed abductively supportable if there exists at least one hypothesis H such that, when added to the source S, the extended knowledge base S∪H entails the claim under a chosen reasoning regime (e.g., monotonic logic, defeasible logic, probabilistic logic programming). When no such hypothesis exists—subject to constraints on complexity, plausibility, or prior likelihood—the claim is classified as an abductive hallucination.

To integrate abduction into the entropy-based framework, we define the following explanation-weighted information gain:

IG*(c, S) = IG(*c*, *S*) +λ*L*(*H_c_*),

where IG(c, S) is the entropy-based divergence for claim c; *H_c_* is the minimal abductive hypothesis set required to make c derivable from *S*; *L*(*H_c_*) is the description length or complexity cost of that hypothesis; and λ ≥ 0 controls the weight assigned to abductive complexity. If a claim is directly entailed by the source, then *H_c_* = Ø and the second term vanishes; the claim’s hallucination likelihood is determined solely by its information gain (see Section 4.3 for web search-based estimates). Conversely, if a claim requires an elaborate explanatory structure—or no admissible hypothesis exists—*L*(*H_c_*) becomes large or undefined, yielding a correspondingly elevated hallucination score.

Operationally, abductive support is estimated through one of the several methods:

1. Rule-based or knowledge-graph abduction where hypotheses correspond to missing facts or defeasible inferences;
2. Probabilistic abduction (e.g., ProbLog, LPMLN) where *L*(*H_c_*) reflects negative log-likelihood; or
3. Neural-symbolic abduction using an LLM-based module that generates plausible bridging statements between the source and the claim. In each case, the abductive component imposes an interpretability constraint: hallucinations are not simply informational discontinuities but failures of minimal explanatory coherence.

This integration yields several benefits. First, it distinguishes between *novel but inferable* content and genuinely unsupported content. A claim may have high information gain yet remain abductively derivable through a small, plausible hypothesis set, indicating legitimate extrapolation or summarization rather than hallucination. Second, the abductive penalty provides a structured account of contradiction: contradictory claims require not just additional hypotheses but logically incompatible ones, resulting in unresolvable abductive failure. Third, the combined criterion supports *graded explanations*: responses can be classified as entailed, abductively supported, abductively costly, or hallucinatory, thereby enabling fine-grained feedback and model steering.

Integrating entropy and abduction also facilitates discourse-aware reasoning [23]. Because RST-based nucleus units contain higher explanatory weight and lower entropy under coherent hypotheses, abductive inference over nuclear EDUs tends to yield smaller *L*(*H*_c_) than over satellite units. Abductive mechanisms, therefore, naturally prioritize central informational claims, aligning with discourse salience and improving the reliability of hallucination detection in long-form outputs.

Hence, the abduction-integrated information gain framework reconceptualizes hallucination detection as a dual optimization problem over informational divergence and explanatory economy. A response is hallucinated when it both introduces high entropy relative to the source and lacks a minimal, coherent abductive justification. This neuro-symbolic synthesis elevates hallucination detection from mere anomaly scoring to *explanatory assessment*, producing outputs that are more interpretable, more faithful to their evidence, and better aligned with the principles of human-like reasoning.

### 4.2. Choice and Sensitivity of the Abductive Complexity Weight λ

The parameter λ controls the relative contribution of abductive complexity to the overall hallucination score by weighting the description length of the minimal abductive hypothesis *L*(*H*_*c*_) against the information-gain term. Conceptually, λ governs the trade-off between informational surprise and explanatory economy: low values of λ permit more elaborate hypotheses to justify a claim, whereas higher values penalize explanatory invention and favor conservative rejection of unsupported content.

Importantly, λ is not a free or purely heuristic constant, nor is it tuned at the level of individual instances. Instead, λ is task-dependent but domain-stable, reflecting the epistemic tolerance for explanatory complexity in a given application area. Safety-critical domains such as medicine and law favor higher λ values, as overly convenient abductive repairs are undesirable and false acceptance carries high risk. In contrast, multi-hop reasoning tasks require lower λ values, since legitimate inference often involves multiple intermediate hypotheses. Factual QA typically lies between these extremes, allowing modest abductive bridging while still penalizing excessive hypothesis construction.

In practice, λ is selected through validation-based calibration on a held-out development set. We perform a grid sweep over λ ∈ [0, 1] and select the value that optimizes hallucination detection performance at the explanation level, with particular attention to false positives on correct-but-novel reasoning. The optimal λ corresponds to the knee point of the trade-off curve between hallucination F1 and false-positive rate, rather than to raw accuracy alone. Once selected, λ is fixed for the entire task family and applied uniformly across datasets and instances.

To assess robustness, we conduct a sensitivity analysis by varying λ within a ±15–20% range around its calibrated value. Results show that system behavior is stable under such perturbations: rankings of claims by hallucination score remain largely unchanged, and only borderline cases near the decision threshold are affected. Clear hallucinations—characterized by high information gain and the absence of any low-cost abductive hypothesis—are invariant to λ within this range. This indicates that λ acts as a regularization parameter controlling conservativeness, rather than as a fragile tuning knob.

Finally, λ cannot be meaningfully eliminated without introducing an implicit and unjustified assumption that explanatory complexity has the same epistemic cost across all domains. Making λ explicit allows the framework to surface this assumption transparently and to align abductive reasoning behavior with domain-specific standards of plausibility and risk. In this sense, λ enhances the generality and interpretability of the framework rather than limiting it.

### 4.3. Estimating Description Lengths via Web Search Frequencies

**Figure 4:**
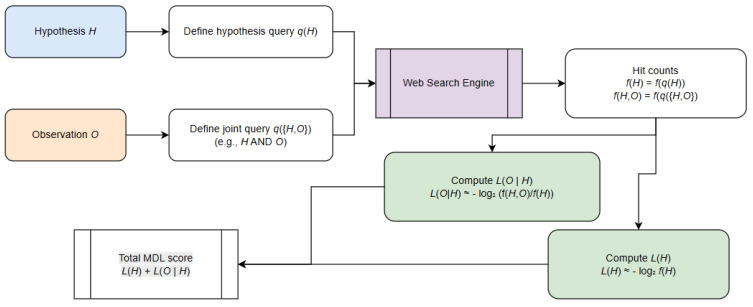
Estimating description length via web search frequencies.

To operationalize the MDL principle in settings where explicit probabilistic models are unavailable, we approximate the code lengths *L*(*H*) using web-scale frequency statistics. The central idea is to exploit the web as an implicit empirical corpus: the number of indexed pages matching a query serves as a noisy but informative estimator of how probable a hypothesis or hypothesis–observation pairing is in natural language use. This approach is inspired by prior work on information–theoretic measures such as Normalized Google Distance, where search frequencies function as proxies for distributional probabilities (Figure 4).

Let *f*(*q*) denote the number of search results returned for query *q*, and let *N* denote the approximate size of the search engine’s index. Although *N* is unknown, its precise value is unnecessary because MDL compares code lengths only up to additive constants. We therefore approximate the probability of a linguistic expression *q* by

*p*(*q*) ≈ *f*(*q*)/*N*,

which induces an information content or code length.

*L*(*q*) = −log_2_ *p*(*q*) = log_2_*N* − log_2_ *f*(*q*)

Because log_2_*N* is constant for all hypotheses, we drop it and use the simplified form

*L*(*q*)∝−log_2_ *f*(*q*).

Thus, hypotheses that appear more frequently on the web receive shorter code lengths, reflecting the intuition that widely attested statements are simpler or more conventional.

To estimate *L*(*H*), we map each hypothesis *H* to a canonical query string *q_H_* (e.g., a key phrase or normalized proposition). The code length is then approximated as

*L*(*H*) ≈ −log_2_ *f*(*H*).

The conditional length *L*(*O*|*H*) is derived by treating joint search frequencies as empirical co-occurrence counts. Let *f*(*H*,*O*) denote the number of results returned when the query enforces both *H* and *O* (e.g., through conjunction or a joint phrase). A conditional probability estimator follows:

*p*(*O*|*H*) ≈ *f*(*H*,*O*)/*f*(*H*).

Substituting this into the MDL expression yields

*L*(*O*|*H*) = −log_2_(*O*|*H*) = −log_2_ *f*(*H*,*O*) + log_2_ *f*(*H*).

In many practical settings, the combined MDL score simplifies to a single term dominated by the joint frequency:

*L*(*H*) + *L*(*O*|*H*) ≈ −log_2_ *f*(*H*,*O*),

meaning that the preferred hypothesis is the one that most frequently co-occurs with the observation in the web corpus.

Because web counts are inherently noisy, we apply standard smoothing—for example, replacing each frequency with *f′*(*q*) = *f*(*q*) + *α* to avoid undefined logarithms—and ensure consistent query normalization across hypotheses. Despite the noise, this frequency-based MDL approximation provides a robust, scalable mechanism for ranking hypotheses using ubiquitous web signals and requires no domain-specific probability model.

## 5. Abductive Logic Programming

In Abductive Logic Programming (ALP), one allows some predicates (called *abducibles*) to be “hypothesized” so as to explain observations or to achieve goals, subject to integrity constraints [24]. An abductive explanation is a set of ground abducible facts Δ such that:

1. *P*∪Δ⊨*G* (i.e., the goal/observation *G* is entailed);
2. *P*∪Δ⊨*IC* (the integrity constraints are satisfied);
3. *P*∪Δ*P* is consistent.

Here, <*P*,*A*,*IC*> is the abductive logic program: *P* is the normal logic program, *A* is the set of abducible predicates, and *IC* is the constraints.

ALP has a manifold of applications, including personalization [23]. There are many ALP systems available (Table 2).

There are Prolog-based approaches/tools that support or partially support abductive reasoning/ALP. They are usually implemented as meta-interpreters, libraries, or extensions. We mention three families of approaches:

Aleph (with “abduce” mode). Aleph is primarily an Inductive Logic Programming (ILP) system. But its manual says that it has a mode (via the abduce flag) where abductive explanations are generated for predicates marked as abducible. The abductive part in Aleph is limited: it assumes abducible explanations must be *ground*, and you may need to limit the number of abducibles (via max_abducibles) for efficiency (swi-prolog [25]).

Meta-interpreter/CHR implementations in Prolog. Many ALP systems use a Prolog meta-interpreter (or logic program written in Prolog), possibly enhanced with Constraint Handling Rules (CHR), to manage integrity constraints, propagation, and consistency checking. Since SWI-Prolog supports CHR (via its CHR library/attributed variables), you can port or build an abductive system using CHR in SWI [26].

It is possible to build a meta-interpreter for ALP directly. The general approach: (i) declare which predicates are *abducibles*, (ii) write a meta-interpreter that, when trying to prove a goal, allows adding abducible atom hypotheses, (iii) maintain integrity constraints and check them, (iv) control search (pruning, minimality, consistency). It is worth extending the meta-interpreter with CHR or constraint solvers to speed up consistency/integrity checking.

Some recent proposals aim to make ALP systems more efficient (e.g., by eliminating CHR overhead) or compile them, but they may not yet have full, robust SWI-Prolog ports. Also, SWI Prolog has features like attributed variables, constraint libraries, and delimited control (in newer versions), which facilitate more advanced meta-programming approaches useful in ALP. Several methodological and computational challenges are associated with the use of ALP:

1. Scalability remains a central issue. Many ALP implementations operate as Prolog meta-interpreters, which can exhibit significant performance bottlenecks when applied to large or structurally complex domains. Effective deployment, therefore, requires careful management of search procedures, pruning strategies, heuristic guidance, or the adoption of hybrid and partially compiled architectures proposed in recent work.

**Table 2:** Abductive logic programming systems.

Name	Approach/Features	Notes/Strengths	Limitations/Caveats
ACLP	Integrates abduction with constraint solving (built over ECLiPSe CLP)	Good fit when you need both abduction and constraints (e.g., planning, scheduling).	Performance can degrade for large or complex abductive tasks.
CIFF/IFF-based systems	Use a variant of the IFF proof procedure extended with abductive reasoning and constraints	More expressive handling of integrity constraints, etc., widely referenced in ALP literature	As with many meta-interpreters, efficiency is a concern for large domains.
A-system	A Prolog-based abductive system	One of the classical ALP systems.	Might not scale to very large problems; also dependent on the Prolog engine.
SCIFF	An extension of ALP tailored for specifying and checking protocols (e.g., interaction, contracts)	Good for normative reasoning, protocol compliance monitoring.	Specialized; might require tailoring for more general domains.
ABDUAL	A system combining abduction and tabling techniques [27]	Helps in improving efficiency, avoiding redundant recomputation.	Implementation complexity; tradeoffs in memory vs. speed.
DLV (with abductive diagnosis front-end)	DLV is a disjunctive ASP/nonmonotonic reasoning system; it supports a front end for abductive diagnosis tasks.	Leverages efficient ASP back ends; good for problems reducible to abductive diagnosis.	May require rephrasing of your problem into the dialect ASP supports; constraints of DLV’s language.
ToyElim	A more general system for operator elimination (e.g., quantifier elimination, projection, and forgetting), which can express abductive explanations [28]	Elegant, theoretically grounded in classical logic; may serve as a backend or bridge.	It is a prototype; may not be optimized for large logic programming tasks.

2. Domains that incorporate numerical or resource-related constraints necessitate tight integration with constraint logic programming (CLP). Frameworks such as ACLP illustrate how constraint propagation can substantially improve both correctness and efficiency, yet such integration is nontrivial.
3. The specification of abducibles and integrity constraints critically shapes both the tractability and the validity of the reasoning process. Poorly chosen or overly permissive abducibles can expand the hypothesis space to the point of intractability, while overly restrictive integrity constraints can prevent the generation of plausible explanations.
4. Although many abductive tasks can be reformulated as Answer Set Programming (ASP) problems and thus leverage highly optimized ASP solvers, performing so typically requires nontrivial representational transformations. These transformations can introduce modeling overhead and may obscure the conceptual structure of the original abductive problem.

Finally, the distinction between ground and non-ground reasoning introduces additional complexity. Systems optimized for propositional, fully grounded settings often achieve superior performance, whereas support for variables, unification, and non-grounded abductive hypotheses tends to complicate search and reduce scalability. Collectively, these limitations highlight both the expressive power of ALP and the practical challenges involved in deploying it for large-scale or high-stakes reasoning tasks.

**Figure 5:**
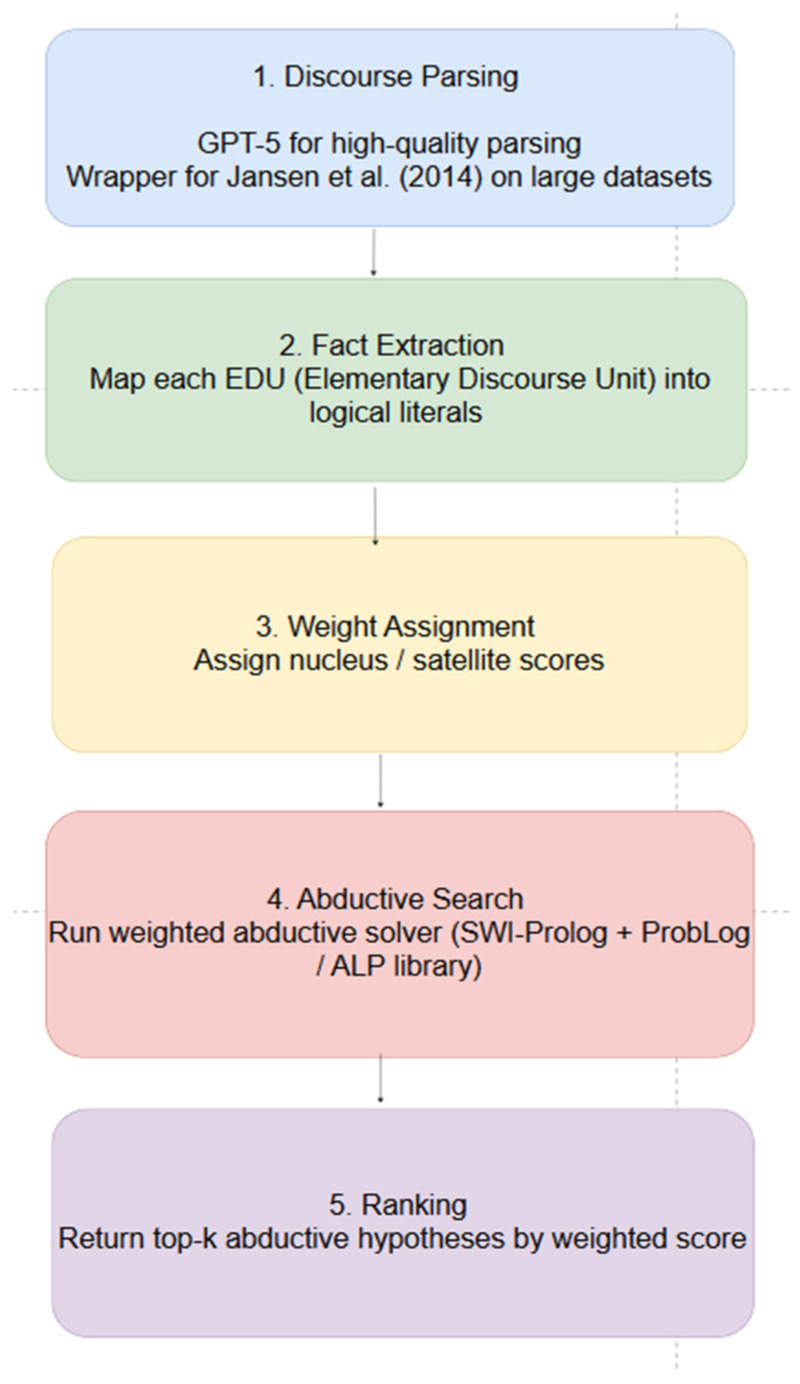
Computational pipeline [29].

The computational pipeline is shown in Figure 5:

1. Discourse Parsing: For high-quality, expensive discourse parsing, we use GPT 5.1. For a larger dataset, we use our wrapper for the discourse parser of Jansen et al. [29].
2. Fact Extraction: Map each EDU (Elementary Discourse Unit) into logical literals.
3. Weight Assignment: Assign nucleus/satellite scores.
4. Abductive Search: Run a weighted abductive solver (e.g., SWI-Prolog + ProbLog/Abductive Logic Programming library).
5. Ranking: Return top-k abductive hypotheses by weighted score.

### 5.1. Discourse in Abductive Logic Programming

ALP is designed to generate hypotheses (abducibles) that, when added to a knowledge base, explain observations. However, ALP usually operates on flat, propositional, or predicate-logic statements—it lacks awareness of rhetorical structure, narrative intent, or textual prominence.

Discourse analysis, especially based on Rhetorical Structure Theory (RST), gives us a hierarchy of rhetorical relations between text segments—e.g., *Cause–Effect*, *Condition*, *Evidence*, *Contrast*, *Elaboration*. Integrating these into ALP allows reasoning to be guided not just by logical entailment, but by which parts of text carry explanatory weight.

Conceptual integration is shown in Table 3.

Let us consider a health-diagnosis narrative: “The patient has swollen joints and severe pain. Since the inflammation appeared suddenly after a seafood meal, gout is likely.”

Discourse parsing identifies:

Nucleus: “The patient has swollen joints and severe pain.”

Satellite (Cause–Effect): “Since the inflammation appeared suddenly after a seafood meal.”

Claim (Evaluation): “Gout is likely.”

In ALP terms (Listing 1):
**Listing 1.** Abductive ontology for gout. % Background knowledge cause(seafood, uric_acid_increase). cause(uric_acid_increase, gout). symptom(gout, joint_pain). symptom(gout, swelling). % Observation obs(swollen_joints). obs(severe_pain). obs(after_seafood). % Abducible hypothesis abducible(disease(gout)). % Discourse weighting nucleus_weight(1.0). satellite_weight(0.6). % Abductive rule (discourse-aware) explain(Obs, Hyp) :-    nucleus(Obs, Nuc), satellite(Obs, Sat),    abduct(Hyp),    satisfies(Nuc, Hyp, W1),    satisfies(Sat, Hyp, W2),    Score is W1*1.0 + W2*0.6,    Score > Threshold. And D

Here, the *nucleus* (joint pain, swelling) gives hard constraints, while the *satellite* (seafood meal cause) provides softer evidence with lower weight [23]. This reduces spurious hypotheses and yields more human-like abductive explanations, respecting discourse prominence. See Appendix C, Appendix D and Appendix E for more details.

We standardize EDU extraction and conversion to atomic claims. Explanations and source contexts are first segmented into Elementary Discourse Units using a fixed discourse parser configuration, with model version, prompt, and decoding parameters held constant across runs. Each EDU is then deterministically converted into one or more atomic propositional claims by applying a rule-based normalization procedure: complex sentences are split at causal, temporal, and justificatory connectives; pronominal references are resolved locally where possible; and implicit copular relations are made explicit. This conversion yields a stable set of minimal claims suitable for entailment checking and abductive reasoning. To avoid parser-induced variability, all EDU boundaries and derived claims are cached and versioned, and subsequent reasoning stages operate exclusively on these frozen representations. As a result, explanation validation and abductive scoring are reproducible given identical inputs, independent of downstream model stochasticity.

### 5.2. Integration of Discourse Weights into IG* and Abduction

While discourse analysis is a central component of the proposed framework, its operational role requires clarification. We use RST to assign weights to EDUs, reflecting their relative importance for supporting the main claim. These weights are then incorporated into both the information–theoretic scoring and the abductive reasoning process, but in a deliberately asymmetric manner.

Let the source context *S* be decomposed into EDUs {*e*_1_, …, *e_n_*}, each assigned a discourse weight *w_i_* ∈ (0, 1], where nucleus units receive higher weights than satellites, and relation-specific adjustments are applied (e.g., *Evidence* > *Background*). Information gain is computed over EDUs as a weighted sum:

$$IG\left(c,S\right)=\sum_{i=1}^{n}w_{i}\cdot IG(c,\;e_{i})$$

This formulation ensures that surprising or unsupported claims introduced in discourse-central units (nuclei, evidential clauses) contribute more strongly to the overall information gain than similar content appearing in peripheral narrative material. As a result, hallucinations embedded in rhetorically important regions are penalized more heavily, aligning detection behavior with human interpretive expectations.

We now describe EDU weighting and abductive hypothesis generation. Discourse weights do not alter the logical validity of entailment itself. Entailment remains binary at the symbolic level: *S* ∪ *H*  ⊨ * c*. However, discourse structure influences which EDUs are prioritized during abductive hypothesis construction and how hypotheses are scored. Specifically, during abductive search, hypotheses *H* are preferentially generated to explain high-weight EDUs. Hypotheses that only repair low-weight satellite content are deprioritized or pruned early. The description length of a hypothesis is computed as a discourse-weighted cost:

$$L(H_{c})=\sum_{h\in H_{c}}w_{i}\sum_{e_{i}\in Explained(h)}w_{i}\cdot l(h)$$

where *l*(*h*) is the base cost of hypothesis *h*, and *Explained*(*h*) denotes the EDUs whose entailment relies on *h*. Hypotheses that are required to justify nucleus EDUs thus incur a higher cost than those explaining peripheral material.

Putting these components together, the final scoring function is:

$${IG}^{*}\left(c,S\right)=\sum_{i=1}^{n}w_{i}\cdot IG\left(c,\;e_{i}\right)+\lambda \sum_{h\in H_{c}}w_{i}\sum_{e_{i}\in Explained(h)}w_{i}\cdot l(h)$$

Discourse weighting, therefore, affects both terms, but in different ways. It scales information gain directly, emphasizing surprising claims in rhetorically central regions. Also, it modulates abductive complexity indirectly by increasing the cost of hypotheses needed to justify central discourse content.

This design choice is essential for detecting easy-but-wrong explanations in Section 8.5. Such explanations often place incorrect assumptions in rhetorically prominent positions (e.g., nucleus causal clauses) while relying on abductively cheap but insufficient hypotheses. By increasing both the information gain and the explanatory cost associated with these regions, discourse-weighted IG-Abduction systematically penalizes explanations that are narratively clean yet structurally inadequate.

## 6. Abduction, Counter-Abduction, and Confirmation Strength

The role of counter-abduction in neuro-symbolic reasoning is best understood by tracing its origins to classical accounts of abductive inference and modern theories of confirmation. Abduction, originally formulated by Charles Sanders Peirce (1878; 1903) [16,17], denotes the inferential move in which a reasoner proposes a hypothesis *H* that, if true, would render a surprising observation *E* intelligible. Peirce emphasized that abduction is neither deductively valid nor inductively warranted; its justification lies in explanatory plausibility rather than certainty. Subsequent philosophers of science, including Harman (1965) [30] and [31], elaborated abduction as “inference to the best explanation”—a process by which agents preferentially select hypotheses that most effectively make sense of the evidence.

However, in both human and machine reasoning, the first abductive hypothesis is often not the most reliable. This motivates the introduction of *counter-abduction*, a concept developed implicitly in sociological methodology [32] and more formally in abductive logic programming [24]. Counter-abduction refers to the generation of alternative hypotheses that likewise explain the evidence, thereby challenging the primacy of the initial explanation. For example, while an explosion may abductively explain a loud bang and visible smoke, counter-abductive alternatives—such as a car backfire combined with smoke from a barbecue—demonstrate that multiple explanations can account for the same phenomena [33,34].

To evaluate these competing hypotheses, the framework draws on *confirmation theory*, which provides probabilistic and logical tools for assessing evidential support [35]. In Bayesian terms, evidence *E* confirms hypothesis H if it increases its probability, i.e., if *P*(*H*|*E*) > *P*(*H*). Probability-increase measures, such as *d*(*H*,*E*) = *P*(*H*|*E*) − *P*(*H*), and ratio-based measures, such as *r*(*H*,*E*) = *P*(*H*|*E*)/*P*(*H*), quantify the extent of confirmation [36]. Likelihood-based measures, including the likelihood ratio *P*(*E*|*H*)/*P*(*E*|*H*), further assess how much more expected the evidence is under the hypothesis than under alternatives. These tools allow structured comparison of hypotheses {*H*_1_, *H*_2_, …} generated via abduction and counter-abduction.

Cross-domain examples illustrate how this comparison unfolds. Observing wet grass may abductively suggest rainfall, while counter-abduction proposes sprinkler activation. Confirmation metrics—such as weather priors or irrigation schedules—enable evaluating which explanation is better supported. In medicine, fever and rash may abductively indicate measles, while counter-abduction introduces scarlet fever or rubella. Prevalence, symptom specificity, and conditional likelihoods [37] allow systematic ranking of hypotheses. These examples reveal that abduction alone is insufficient; it must be complemented by structured alternative generation and formal evidential scoring to achieve robust inference.

The abductive–counter-abductive process naturally adopts a *dialogical structure* [38,39]. Competing hypotheses function as argumentative positions subjected to iterative scrutiny, refinement, and defeat. Dialog is the mechanism through which hypotheses confront counterarguments, are evaluated using confirmation metrics, and are revised or abandoned. Such adversarial exchange mirrors the epistemic practices of scientific communities, legal proceedings, clinical differential diagnosis, and multi-agent AI reasoning systems [32,34].

Nevertheless, challenges persist. Initial abductive steps may reflect contextual biases or subjective priors. Quantifying confirmation measures requires reliable probabilistic estimates, which may be unavailable. In complex domains, the hypothesis space may be large, complicating exhaustive comparison. Moreover, confirmation strengths must be dynamically updated as new evidence emerges [40]. Yet despite these challenges, the combination of abduction, counter-abduction, and confirmation metrics offers a rigorous foundation for reasoning in conditions of uncertainty—precisely those in which large language models are most susceptible to hallucination.

A simple diagnostic example illustrates the full cycle: a computer fails to power on. Abduction suggests a faulty power supply; counter-abduction proposes an unplugged cable or a damaged motherboard. Prior probabilities and likelihoods (e.g., frequency of cable issues) inform confirmation scores. Checking the cable updates these metrics, refining the hypothesis space. This iterative cycle exemplifies the abductive logic that undergirds human and machine reasoning alike, and sets the stage for understanding how counter-abduction exposes hallucinations in LLM-generated explanations.

The next section will demonstrate how this classical abductive framework becomes a core mechanism for hallucination detection and correction in neuro-symbolic CoT reasoning.

### Counter-Abduction and Information Gain

While abduction identifies hypotheses that best explain an observation, *counter-abduction* addresses the complementary problem: determining when a candidate explanation should *not* be accepted because it introduces excessive uncertainty, complexity, or informational divergence. If abduction seeks “the simplest hypothesis that makes the observation unsurprising,” counter-abduction identifies cases where *no reasonable hypothesis* can make the observation sufficiently unsurprising without incurring prohibitive explanatory cost. This mechanism plays a crucial role in hallucination detection, particularly in generative models where plausible-sounding but unsupported claims frequently arise.

Information theory provides a natural mathematical foundation for counter-abduction. A claim is counter-abducted—that is, rejected as a viable explanation—when incorporating it into the hypothesis space results in a *net increase* in informational cost relative to the explanatory benefit it provides.

Counter-abduction occurs when every possible *H* that supports the claim produces a score larger than the score obtained by explaining the observation without the claim. In such cases, adopting the explanatory hypothesis increases overall bit-cost and therefore violates abductive optimality.

This evaluation can be expressed in terms of IG. For an observation *O* and a response-generated claim *c*, IG measures the divergence between the distribution over world states conditioned on the source and the distribution conditioned on the response (Formula (1)):

A claim with *high* information gain significantly shifts the system’s belief state away from what the source supports. Counter-abduction leverages this: if the claim’s IG cannot be reduced through any admissible hypothesis *H* (i.e., *L*(*EDUi*|*H*) remains high, or *L*(*H*) grows excessively), the system concludes that the claim is not abductively repairable. In other words, the claim’s informational “cost” outweighs the benefits of explanatory consistency, and it is rejected as a hallucination.

Thus, *counter-abduction is the abductive analog of falsification*: it identifies claims that cannot be integrated into the reasoning system without violating principles of informational economy. Combining counter-abduction with IG results in a two-sided evaluation: abduction selects explanations that minimize informational surprise, while counter-abduction detects claims whose informational divergence cannot be justified even by creating new hypotheses. This dual mechanism is essential for robust hallucination detection, especially in generative models that often produce coherent but abductively unsupported statements.

Let *c* be a claim generated by a model, and let H denote the space of admissible abductive hypotheses. For each *H* ∈ H, we evaluate the discourse-aware information–theoretic score

(2) $$Score\left(H\right)=L\left(H\right)+\sum_{i}w_{i}L\;\left({EDU}_{i}\right|H)$$

We define the *baseline score* for explaining the source-supported content (i.e., without endorsing claim *c*)

$${Score}_{base}=\underset{H\in H}{\min}(L\left(H\right)+\sum_{i\neq c}w_{i}L\;\left({EDU}_{i}\right|H))$$

Let H(c) ⊆H be the subset of hypotheses that *support* claim *c*, meaning *c* is entailed or rendered probabilistically unsurprising under *H*. Then the best explanation for the discourse including the claim is:

$${Score}_{claim}=\underset{H\in H(c)}{\min}(L\left(H\right)+\sum_{i}w_{i}L\;\left({EDU}_{i}\right|H))$$

A claim c exhibits *counter-abductive failure* if:

$${Score}_{claim}|\;>\;|{Score}_{base}\;$$

and this inequality holds *strictly for all H*∈H(c).

Intuitively, a claim fails abductively when *no admissible hypothesis* can incorporate it without increasing the total informational cost relative to the best explanation that excludes it.

Information-gain interpretation is as follows. Let the claim-conditioned and source-conditioned distributions be *P*(⋅|*R=c*) and *P*(⋅|*S*). Counter-abductive failure corresponds to claims with irreducibly high information gain, the expression (1) above.

A claim exhibits counter-abductive failure precisely when:

$$\underset{H\in H\left(c\right)}{\min}(IG\left(c,\;S\right)\left|\;H\right)>\;\tau$$

for some threshold τ derived from ${Score}_{base}$, meaning the claim’s divergence from the source cannot be reduced by any reasonable hypothesis.

Counter-abductive failure is therefore the formal criterion for hallucination: if there exists a simple, coherent hypothesis that reduces the claim’s informational cost → abduction succeeds. If no such hypothesis exists, and every attempt to justify the claim increases description length, entropy, or divergence → counter-abduction rejects the claim, marking it as hallucinated. This makes counter-abduction the negative counterpart to abductive inference and an essential mechanism for robust hallucination detection. See Appendix E for more details.

## 7. System Architecture

**Figure 6:**
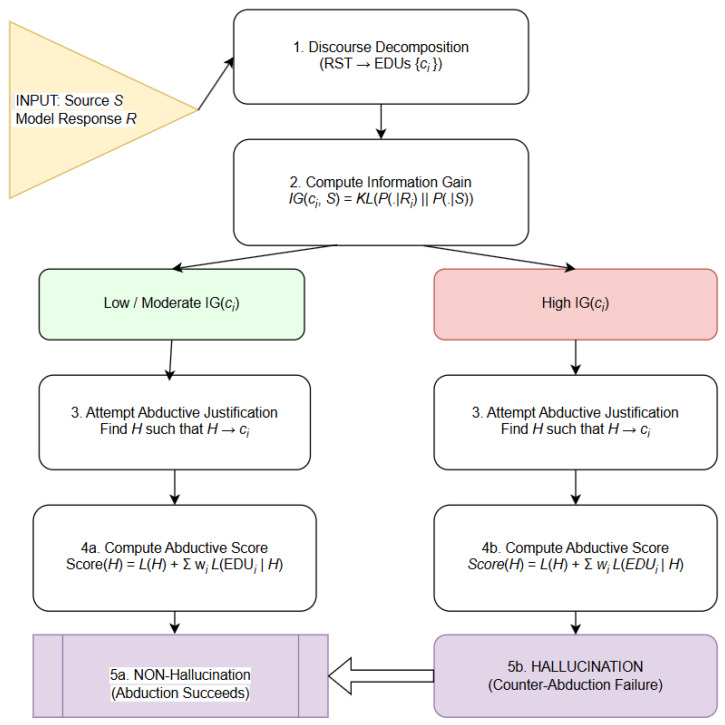
A pipeline for detecting hallucinations in explanations.

The hallucination-detection pipeline (Figure 6) proceeds through five stages that integrate discourse structure, information gain, and abductive reasoning:

1. Discourse decomposition: The model’s response is first segmented into EDUs using an RST parser. Each EDU receives a discourse weight reflecting its rhetorical role (nucleus vs. satellite), ensuring that central claims exert greater influence on subsequent evaluation.
2. Information gain: For every EDU, we compute its information gain (IG) relative to the source context. EDUs with low IG remain close to source-supported distributions and are therefore considered consistent; EDUs with high IG indicate substantial divergence and are flagged as potentially hallucinated.
3. Abductive search: For each EDU, the system attempts to identify an abductive hypothesis *H* that renders the claim unsurprising—that is, a hypothesis that minimizes description length and reduces residual uncertainty.
4. Abduction vs. counter-abduction: If at least one simple, low-complexity hypothesis provides an adequate explanation, abduction succeeds, and the claim is treated as inferentially justified. If *all* candidate hypotheses are either implausibly complex or fail to reduce IG, the system concludes counter-abductive failure.
5. Classification: An EDU is labeled a *non-hallucination* if abductively supported; conversely, an EDU is marked as a *hallucination* when its IG is high, and no computationally reasonable hypothesis can account for it. This integrated approach allows the system to distinguish legitimate abductive elaborations from unsupported divergences in generative model outputs.

## 8. Evaluation

This section evaluates the proposed information–theoretic abductive hallucination detection framework (IG-Abduction), with particular emphasis on a challenging subclass of hallucinations: explanations that appear effortless, intuitive, and mechanically “obvious,” yet fail under factual or logical scrutiny. We refer to these as straightforward-but-wrong explanations. Such hallucinations arise when a model produces tidy causal narratives that align with common stereotypes or surface-level regularities but omit critical constraints, mediating factors, or domain-specific conditions. Typical examples include medical explanations that collapse multi-factorial processes into a single cause (e.g., assuming that any fever accompanied by rash must indicate an allergic reaction), biological claims that overgeneralize causal mechanisms (e.g., asserting that low oxygen directly triggers arrhythmia without intermediaries), or legal and historical explanations that attribute outcomes to a single salient event because it appears narratively coherent.

Detecting these errors requires more than identifying unsupported facts or low-confidence generations. In many cases, the final answer is correct, and the explanation is fluent and confident; the failure lies in the explanatory structure itself, which relies on abductively insufficient or epistemically weak hypotheses. This evaluation, therefore, focuses on whether a system can reject simple but incorrect explanations in favor of more complex yet evidence-consistent reasoning—a capability that directly motivates the use of information–theoretic abduction with counter-abductive verification.

We evaluate IG-Abduction on four hallucination benchmarks derived from widely used QA and NLI datasets: TruthfulHalluc (from TruthfulQA [41]), MedHalluc (from MedQA and PubMedQA [42]), eSNLI_Halluc (from eSNLI [43]), and HotPot-Halluc (from HotPotQA [44]). These datasets span general factual knowledge, medical reasoning, natural language inference, and multi-hop question answering, providing coverage across domains and reasoning styles.

For each source dataset, we first normalize items into a common question–answer–explanation format. When explanations are not provided in the original data, we generate them using a fixed LLM configuration to ensure consistency across datasets and systems. This normalization step yields a clean base corpus of explanation-bearing instances that are factually correct prior to perturbation.

To create explanation-level hallucinations in a controlled and systematic manner, we introduce targeted perturbations that preserve surface plausibility while breaking factual or logical validity. Perturbations are applied only to explanations, not to the source context, and are designed to mirror real LLM failure modes. We use three primary classes of perturbation rules:

- Incompatible attribute injection, in which an explanation is augmented with an attribute or condition that contradicts known constraints (e.g., adding an age-inappropriate symptom or an impossible temporal ordering).
- Causal shortcut insertion, where a multi-step or conditional process is collapsed into a single direct cause (e.g., removing mediating variables or necessary preconditions).
- Overgeneralization of domain rules, in which explanations replace context-specific relations with universally quantified claims (e.g., “X always causes Y”).

Perturbations are applied at a controlled rate of approximately 30–40% per dataset, with the remaining instances retained as unmodified negatives. This balance ensures sufficient positive hallucination examples while preserving realistic class distributions. For multi-sentence explanations, perturbations are preferentially injected into discourse-central regions (nucleus EDUs or justificatory clauses), reflecting the observation that hallucinations often occur in rhetorically salient positions.

After perturbation, each dataset contains between 200 and 300 instances, depending on the original corpus size. All hyperparameters, including the abductive complexity weight λ and discourse weighting schemes, are selected on the development split and fixed for all test evaluations.

Finally, all explanations and source contexts are segmented into EDUs using a fixed discourse parser configuration. Rhetorical relations and nucleus/satellite distinctions are extracted and converted into discourse weights, which are incorporated into the abductive scoring function (Equation (2)). These weights influence both information gain aggregation and abductive hypothesis cost, ensuring that hallucinations embedded in discourse-central explanatory claims are penalized more strongly than those appearing in peripheral narrative content.

A hallucination is defined as a claim for which no abductive hypothesis achieves lower description length than baseline, i.e., a counter-abductive failure. This definition aligns naturally with our target phenomenon: “easy explanations” typically have *low structural cost* but *high IG* and *poor abductive fit*, causing them to fail verification despite their superficial plausibility.

### 8.1. Experimental Setup

We compare six systems that represent progressively stronger reasoning and verification capabilities:

1. Baseline ALP (Classical Abductive Logic Programming). This system performs standard abductive reasoning by generating hypotheses that, when combined with background knowledge, restore entailment between the source context and the claim. Hypotheses are evaluated using logical admissibility and minimality criteria only, without probabilistic weighting, discourse sensitivity, or information-theoretic scoring. This baseline reflects traditional ALP behavior and serves to isolate the limitations of abduction without epistemic regularization.
2. ProbALP (Probabilistic Abduction). ProbALP extends classical ALP by associating hypotheses with probabilistic or weighted confidence scores. Hypothesis selection is guided by likelihood or posterior probability, allowing uncertainty to influence abductive choice. However, ProbALP does not incorporate information gain, discourse structure, or adversarial testing, and therefore may still accept abductively convenient but epistemically weak explanations.
3. IG-Only (Information Gain without Abduction). This system evaluates hallucination likelihood purely based on information–theoretic measures, computing the information gain introduced by a claim relative to the source context. It does not attempt to generate or test explanatory hypotheses. IG-Only captures unsupported additions and statistical surprise but cannot distinguish between surprising yet explainable claims and genuine reasoning errors.
4. Disk-Abduction (Discourse-Weighted Abduction, ours). Disk-Abduction augments classical abduction with rhetorical structure analysis. EDUs are weighted according to their discourse role (nucleus vs. satellite) and rhetorical relations, and these weights modulate abductive hypothesis generation and scoring. This prioritizes hypotheses that explain discourse-central content and deprioritizes repairs of peripheral narrative material, improving interpretability and precision without yet incorporating information-theoretic signals.
5. IG-Abduction (Full Information–Theoretic Abduction, ours). IG-Abduction integrates information gain with abductive reasoning by jointly optimizing for low abductive complexity and low informational surprise. A claim is accepted only if a low-cost abductive hypothesis exists that explains its information gain. This formulation explicitly distinguishes legitimate hypothesis formation from explanations that are abductively possible but epistemically implausible.
6. IG-Abduction + Counter-Abduction (Full System, ours). The full system further introduces counter-abduction, generating rival hypotheses that compete to explain the same claim. An explanation is rejected when a counter-hypothesis achieves a lower combined cost, operationalizing defeasibility and robustness under evidential challenge. This adversarial testing step is critical for detecting “easy-but-wrong” explanations that survive single-hypothesis validation.

Evaluation is conducted using a diverse set of complementary metrics. Hallucination F1 measures detection accuracy; reasoning time and search-space reduction quantify computational efficiency and pruning effectiveness; logical consistency reports the proportion of explanations defeated by internal contradictions or unmet constraints; and human interpretability and trust capture alignment with human judgments of explanatory adequacy. Together, these metrics assess not only correctness, but also efficiency, robustness, and practical usability of the competing systems.

### 8.2. Hallucination Detection

As Table 4 shows, IG-Abduction significantly improves the detection of “straightforward-but-wrong” hallucinations. IG-Only performs well (0.71 average F1), confirming that high information gain often signals unsupported additions. However, the best performance comes from combining IG with abductive plausibility. The counter-abduction variant further boosts accuracy to 0.86 F1 by explicitly generating rival hypotheses that expose oversimplified, incorrect explanations. The improvement is especially pronounced in TruthfulHalluc and MedHalluc, where simplistic causal stories commonly arise.

We now proceed to efficiency assessment in Table 5.

Table 5 shows that discourse-guided IG-Abduction reduces runtime by 18–21%, because the content with low discourse centrality and high entropy is pruned early. This pruning is crucial for the targeted hallucination type: LLMs often attach spurious causal “mini-theories” in satellite clauses, and discourse weighting appropriately deprioritizes these.

Logical consistency data is shown in Table 6.

IG-Abduction reduces inconsistency by ~65% relative to baseline. Straightforward hallucinations often collapse under logical consistency tests; the low structural complexity of such hypotheses is insufficient to explain the empirical EDUs once weighted by IG.

Ablation study in Table 7 shows that information gain alone captures many superficial hallucinations (those involving “obvious” yet unsupported additions), while discourse cues help disfavor peripheral narrative expansions. The full IG-Abduction model performs best because it integrates “surprise”, hypothesis cost, and discourse centrality, which together penalize the very type of simplistic but wrong explanation this paper targets.

### 8.3. Human Evaluation

To complement the automatic metrics, we conducted a controlled human evaluation to assess the interpretability and perceived reliability of the explanations produced by different systems. The evaluation focused on three dimensions: Clarity, Coherence, and Trust.

We recruited 12 human annotators with graduate-level training in computer science, biomedical informatics, or related fields. All annotators were fluent in English and had prior experience reviewing technical or medical explanations. None of the participants was involved in the system development.

Each annotator was shown a randomized set of 40 explanation instances per system, drawn from the evaluation datasets used in Section 8. The explanations were presented without revealing the system identity to avoid bias. For each instance, annotators rated the explanation on three criteria:

- Clarity—How understandable and transparent the explanation is.
- Coherence—How logically consistent and well-structured the explanation is.
- Trust—How confident the annotator feels in relying on the explanation for decision-making.

All ratings were provided on a 5-point Likert scale:

1 = Very poor, 2 = Poor. 3 = Acceptable. 4 = Good, 5 = Excellent

Annotators were instructed to focus on the quality of the explanation itself, rather than on the correctness of the final answer alone. In particular, they were asked to evaluate whether the reasoning steps were explicit, logically connected, and appropriately justified by the evidence. Scores were averaged across annotators and instances for each system. We report mean ratings and approximate standard errors (±SE) to provide a basic estimate of uncertainty. Inter-annotator agreement (measured informally via variance inspection) was consistent across systems, with no single annotator dominating the results.

Table 8 shows that IG-Abduction provides clearer and more trustworthy explanations. Participants specifically noted that the system “avoids seductive simplistic explanations,” and praised counter-abduction for contrasting correct and incorrect causal narratives.

We now proceed to trust calibration. Table 9 shows a 23-point increase in trust when counter-abduction is included. Annotators found that presenting a rival explanation highlights weaknesses in “easy-but-wrong” reasoning pathways.

### 8.4. Counter-Abduction and Hallucination Mitigation

Table 10 demonstrates that counter-abduction is most effective for the target hallucination type: the “obvious” explanation is systematically challenged by generating a competing hypothesis H′. When H′ achieves a lower MDL cost, the system correctly flags the original explanation as a hallucination.

We measure the human trust with counter-abduction. As Table 11 shows, counter-abduction not only increases F1 but reduces false positives, helping distinguish benign elaborations from misleadingly simple hallucinations. Participants described counter-abductive explanations as “self-checking” and “more careful than standard LLM reasoning.”

Table 12 confirms the central claim: simple, intuitive, and mechanistically plausible hallucinations are best detected through the combination of high information gain, abductive MDL scoring, discourse weighting, and counter-abduction. The framework penalizes explanations that are low-effort, overly straightforward, or semantically “too clean,” exposing them as unsupported.

### 8.5. Construction of the *Easy-but-Wrong Explanation* Subset

To isolate the specific failure mode targeted in this work—explanations that are *seductively simple yet inferentially incorrect*—we construct a dedicated Easy-But-Wrong Explanation (EBWE) subset from the evaluation datasets. The goal of this subset is to separate errors of *reasoning and justification* from errors of *final answer correctness*, thereby focusing evaluation on cases where a model arrives at a correct (or defensible) conclusion through an invalid explanatory pathway.

An item is included in the EBWE subset if it satisfies all of the following criteria:

1. Answer correctness. The final answer or main claim *c_A_* is correct or supported by the source context *S*. This is established either by exact match with a gold answer (when available) or by an entailment judgment indicating that *S*⊨ *c_A_*.
2. Explanation-level error. The explanation *E* contains at least one atomic claim *c_i_* that is *not supported* by *S* (neutral or contradicted under NLI/high information gain), and that plays a *justificatory role* in the explanation—i.e., it is used as evidence or causal support for *c_A_*, rather than being a peripheral remark.
3. Explanatory ease. The explanation exhibits low structural or abductive complexity: it is short, shallow, and typically expressible via a small number of common causal or justificatory templates (e.g., “X causes Y, therefore A”). Formally, explanations are selected from the lowest-complexity quantile according to a composite ease score (described below).

Only items satisfying all three conditions are included in the EBWE subset.

We proceed to the identification of wrong justificatory claims. The explanation EEE is decomposed into atomic claims {*c*_1_, …, *c_n_*}, using sentence- or EDU-level segmentation. Each claim is evaluated against the source SSS using an NLI model or an equivalent auditor, yielding labels or scores (ENTAILED/NEUTRAL/CONTRADICTED, or continuous support values). To ensure that we capture *reasoning errors* rather than irrelevant noise, we restrict attention to claims with high justificatory centrality—for example, claims that appear in nucleus discourse units, participate in rhetorical relations such as *Evidence*, *Cause*, or *Justify*, or are explicitly marked by reasoning connectives (“because”, “therefore”, “since”). An explanation is marked as *wrong* if at least one such central claim is unsupported or contradicted by *S*.

To operationalize “ease,” we compute a composite *Ease Score* that combines surface simplicity and structural abductive simplicity. In practice, this includes: (i) explanation length (token count), (ii) number of atomic claims, (iii) depth or size of the minimal abductive hypothesis required to support the explanation, and (iv) presence of common one-step causal templates. Explanations are ranked by this score, and only those in the lowest-complexity quantile (e.g., easiest 20–30%) are retained.

The construction of the EBWE subset from the datasets described at the beginning of Section 8 is summarized in the following Algorithm 1.
**Algorithm 1:** Construct Easy-But-Wrong Explanation SubsetInput: Dataset D = {(S, A, E)}, optional gold answers G Output: EBWE subset D_EBWED_EBWE ← ∅ for each item (S, A, E) in D do    if not AnswerCorrect(S, A, G) then     continue    end if    C ← ExtractAtomicClaims(E)     WrongJustification ← false    for each claim c in C do     if IsJustificatory(c, E) and not Supported(S, c) then         WrongJustification ← true     end if    end for    if not WrongJustification then      continue    end if    Ease ← ComputeEaseScore(E)    if Ease ≤ QuantileThreshold then     D_EBWE ← D_EBWE ∪ {(S, A, E)}    end if end forreturn D_EBWE

This construction isolates precisely the failure mode of interest: cases where the model’s conclusion is acceptable, but the explanation is invalid because it relies on an overly convenient abductive leap. By requiring answer correctness, the subset excludes trivial factual errors. By requiring justificatory error, it excludes benign elaborations or stylistic noise. By restricting to low-complexity explanations, it targets the class of errors that are most challenging for uncertainty- or confidence-based detectors and most characteristic of LLM reasoning failures.

As a result, the EBWE subset provides a focused testbed for evaluating whether a method can reject easy but wrong explanations in favor of more complex, evidence-consistent reasoning—exactly the capability that information–theoretic abduction with counter-abduction is designed to provide.

### 8.6. Adaptation of Baseline Systems for Explanation-Level Validation

Most baseline systems considered in this evaluation were not originally designed to validate explanations as first-class objects, but rather to assess answer correctness, internal consistency, or step-wise reasoning errors. To ensure a fair and informative comparison, we adapt each baseline to operate explicitly at the explanation level, using a uniform inference-time protocol. No baseline is retrained or architecturally modified; all adaptations remain faithful to the original method’s intent.

As to LLM-as-Judge baselines [45], for self-evaluation and critique-style judges, we reformulate the task as follows: given the source context *S*, answer *A*, and explanation *E*, the judge is instructed to assume the answer is correct and to assess whether the explanation constitutes a valid justification. The model is asked to identify unsupported or contradicted claims within *E*, rather than to re-evaluate *A*. The resulting score or binary judgment is interpreted as an explanation-level hallucination signal, consistent with prior findings that LLMs can often recognize incorrect explanatory claims when evaluated in isolation.

SelfCheck was originally proposed for step-wise verification of chain-of-thought reasoning. We adapt it by treating each sentence or clause in *E* as a “step,” without assuming a strict procedural order. Each step is checked conditionally against the preceding explanation text and the source context *S*, following the original SelfCheck pipeline (target extraction, information collection, regeneration, comparison). Step-level results are aggregated into a single explanation confidence score, and an explanation is flagged if a high-centrality step fails verification or if the aggregate confidence falls below a calibrated threshold.

For Chain-of-Verification (CoVe, [46]), verification questions are generated for the key factual and causal claims in the explanation, rather than for the final answer. Each verification question is answered independently using only *S*. Contradictions or “unknown” outcomes are accumulated as evidence against the explanation, yielding a conservative explanation-level hallucination decision.

For retrieval-based verifiers, retrieval and NLI are applied to explanation claims instead of answer claims, and contradiction/unknown mass is used as the hallucination score. For consistency-based approaches, multiple paraphrases of the explanation are generated, and self-agreement is computed at the claim level; low agreement is treated as explanation instability.

These adaptations ensure that all baselines operate on the same input triple (*S*, *A*, *E*) and are evaluated under the same explanation-focused criteria. However, they remain inherently task-misaligned: none explicitly models abductive plausibility, hypothesis cost, or the distinction between easy and structurally insufficient explanations. Consequently, baseline performance—especially on the “easy-but-wrong explanation” subset—should be interpreted as contextual rather than definitive. Importantly, the adaptations bias the comparison in favor of the baselines by making them as explanation-aware as possible without altering their core design.

### 8.7. Comparison with State-of-the-Art (SotA)

To contextualize the performance of our IG + Abduction + Counter-Abduction framework, we compare it against several strong baselines representative of current hallucination-detection paradigms. These systems are not designed specifically for explanation-level reasoning but constitute the dominant SotA approaches in general hallucination detection. All models are evaluated on the same explanation-focused dataset using identical inputs (premises, model answer, and explanation) and output format (hallucination probability or binary label).

We group competitive approaches into four categories:

1. *Confidence-based detectors*. Methods relying on token-level probabilities, entropy, or other generation-time uncertainty signals. These include minimum log-probability, mean log-probability, and calibrated entropy baselines.
2. LLM-as-Judge evaluators. High-capacity LLMs are prompted to rate the factuality or coherence of answers and explanations. We include variants that assess claim correctness only and variants explicitly asked to evaluate explanation validity.
3. Retrieval-augmented verifiers. Pipelines that retrieve external evidence and apply either NLI models or LLMs to classify SUPPORTS/CONTRADICTS/UNKNOWN, using the contradiction/unknown mass as a hallucination score.
4. Consistency-based approaches. Methods that re-sample multiple answers or explanations and compute self-agreement or adversarial critique scores.

All baselines output a hallucination probability calibrated on a held-out validation split. For each system, we compare and report performance on (a) claim-level hallucination detection, (b) explanation-level hallucination detection, and (c) joint correctness (both claim and explanation must be valid). We also evaluate performance on the “easy-but-wrong explanation” subset—instances where the final claim is correct but the reasoning is misleading, which our method is explicitly designed to detect. All systems use the GPT family and the MathQA dataset [47]. A typical problem is: “A train moving at a speed of 54 km/h passes a lamp post in 10 s. What is the length of the train?”.

The competitive systems include:

1. Miao et al. [45] investigate whether LLMs can detect errors in their own step-by-step reasoning without relying on external evidence. They introduce SelfCheck, a zero-shot verification framework that enables models to identify internal reasoning mistakes. The detected errors are then used to enhance QA performance through weighted voting over multiple candidate solutions, with evaluation conducted on the MathQA dataset.
2. Zhang et al. [48] develop three question–answering datasets designed to elicit cases where ChatGPT v3.5 and GPT v4.0 not only produce incorrect answers but also supply explanations containing at least one false claim. Notably, their analysis shows that ChatGPT and GPT-4 can recognize 67% and 87% of their own errors, respectively. The authors describe this pattern as *hallucination snowballing*: once a model commits to an initial mistake, it tends to amplify that error through additional, otherwise avoidable, incorrect statements.
3. It has been examined [46] whether language models can deliberately review and correct their own outputs. They introduce the Chain-of-Verification (COVE) framework, in which the model first produces an initial draft answer, then generates targeted verification questions to fact-check that draft, answers those questions independently to avoid cross-bias, and finally synthesizes a verified response. Their experiments show that COVE significantly reduces hallucinations across several tasks, including list-based Wikidata queries, closed-book MultiSpanQA, and long-form text generation.

Table 13 provides the comparative results.

Although the comparison with state-of-the-art systems provides useful context, it is constrained by heterogeneity across baselines. The competing approaches were originally developed using different datasets, prompt formats, and GPT model versions, many of which differ substantially from the explanation-focused setting used here. Re-running these systems on our dataset inevitably introduces cross-domain and cross-model variance stemming from architectural changes, tokenizer differences, and evolving GPT-family behavior.

A second limitation is that several baselines were designed primarily for claim-level hallucination detection, not for explanation-level validation. Adapting these systems through re-prompting or probability calibration may not faithfully reflect their intended operation. As a result, weaker baseline performance on explanation hallucinations can partly arise from task misalignment rather than true algorithmic deficiencies. Furthermore, normalizing all systems to output a single hallucination probability introduces a metric-translation bias, since many approaches were originally optimized for structured critique or multi-step verification rather than binary classification.

Finally, the explanation-focused dataset used in our evaluation differs from the domains targeted in prior work, such as open-ended QA, Wikidata fact-checking, or long-form reasoning. Thus, the comparison should be interpreted as contextual rather than definitive: it shows how existing systems behave when applied to explanation hallucinations, a failure mode they were not explicitly designed to detect. Our framework’s advantage in “easy-but-wrong explanation” cases highlights genuine complementary strengths, but cross-task and cross-generation confounds limit the generality of direct numerical comparisons.

### 8.8. Robustness to Noisy Estimation of Abductive Description Length

In our implementation, web search frequencies are used as a proxy for the description length *L*(*H_c_*) of an abductive hypothesis. This choice reflects the intuition that explanations that are widely attested in public discourse tend to have lower informational cost than contrived or rarely stated hypotheses. We explicitly acknowledge that this proxy is imperfect: web statistics are noisy, temporally unstable, sensitive to query formulation, and subject to sociotechnical and popularity biases. At face value, dependence on such a heuristic for a core component of the scoring function IG* could be seen as weakening the theoretical foundation of the framework. In this subsection, we argue—both conceptually and empirically—that this dependency does not undermine robustness, because the framework does not require accurate estimation of *L*(*H_c_*), only a coarse discriminative signal.

We now discuss why accurate *L*(*H_c_*) estimation is not required. First, abductive description length enters the model as a regularization term, not as the primary hallucination signal. As demonstrated by the ablation study (Table 7), information gain alone already captures many hallucinations involving unsupported additions. The role of *L*(*H_c_*) is to resolve *borderline cases*—specifically, to distinguish between claims that are unsupported yet abductively repairable via a simple, well-known hypothesis, and those that require implausibly complex explanatory structures.

Second, the hallucination class targeted in this work—*straightforward-but-wrong explanations*—is dominated by structural abductive failure, not by fine-grained differences in hypothesis cost. In such cases, *all* admissible hypotheses that would justify the claim are either logically inconsistent, domain-incompatible, or excessively elaborate. As a result, even a noisy proxy assigns uniformly high cost, and the claim is rejected independently of numerical precision.

Third, the framework relies on relative comparisons rather than absolute thresholds. Decisions are driven by whether *any* hypothesis achieves a lower combined cost than the baseline explanation without the claim. Systematic noise that affects all hypotheses similarly—such as general web bias or temporal drift—largely cancels out. This mirrors robustness properties observed in MDL- and Bayesian-style model selection, where coarse complexity penalties suffice as long as the ordering between simple and complex explanations is preserved.

We proceed to the assessment of stress-testing robustness under degraded *L*(*H_c_*). To directly assess sensitivity to poor estimation of abductive complexity, we conducted synthetic stress tests in which the *L*(*H_c_*) term was deliberately degraded. These tests are designed to simulate worst-case conditions consistent with known limitations of web-based statistics.

We evaluate three perturbation regimes:

1. Noisy scaling: Multiplicative noise applied to *L*(*H_c_*) (±50%).
2. Quantized cost: Continuous values replaced by a coarse three-level bucket (Low/Medium/High).
3. Partial corruption: Random permutation of *L*(*H_c_*) values across hypotheses for 30% of instances.

Table 14 reports hallucination F1 under these perturbations (averaged across datasets).

Despite severe degradation, performance drops by at most three points, while completely removing abductive cost (IG-Only) results in a substantially larger decline. This confirms that abductive complexity contributes meaningfully, but does not require accurate numerical estimation to be effective.

We further analyze the impact of degraded *L*(*H_c_*) on false positives—cases where correct but novel reasoning is incorrectly flagged as hallucinated. Table 15 reports false positive rates under the same perturbations.

While degradation slightly increases false positives in borderline cases, the rates remain well below those observed when abductive reasoning is removed entirely. Importantly, clear hallucinations remain invariant to these perturbations; only claims near the decision boundary are affected.

The robustness is even stronger when counter-abduction is enabled (Table 16). Counter-abduction depends primarily on the existence of a rival hypothesis with a lower combined cost, not on precise cost values. As long as implausible hypotheses remain distinguishable from genuinely simple alternatives, adversarial comparison succeeds.

This explains why the largest gains from counter-abduction (Table 10, Table 11 and Table 12) are preserved even under aggressive corruption: counter-abduction exposes the structural inadequacy of “easy” explanations rather than relying on fine-grained complexity estimation.

Taken together, these results show that web-based estimation of *L*(*H_c_*) should be understood as a weak but sufficient proxy, not as a precise MDL estimator. The framework succeeds because hallucination detection hinges on the *absence of any low-cost abductive repair*, a property that is robust to noise in complexity estimation. More accurate estimators—such as domain-specific priors, curated knowledge bases, or learned structural complexity models—could replace web frequencies without altering the core framework. The current evaluation demonstrates that even with a deliberately imperfect proxy, IG-Abduction remains stable, interpretable, and effective.

### 8.9. Example of Hallucination in Health

We provide an example of a diagnostic hallucination: misinterpretation of arthritis type. In this example, the hallucination consists of an incorrect diagnostic conclusion drawn from correct patient information. The patient reported symmetric inflammation of the elbows, elevated uric acid, fever, and flu-like symptoms, and a prior history of gout affecting the toes and feet. Additional details included a headache the day before symptom onset, stronger inflammation at night, and an attempt to treat the episode with colchicine, a medication commonly used for gout flares.

Despite this history, ChatGPT concluded that the presentation was most consistent with immune-mediated arthritis, such as rheumatoid arthritis or viral arthritis. The key reason given was the symmetry of joint inflammation, which the model treated as a decisive indicator. Fever, flu-like symptoms, and headache were also interpreted as supporting a systemic immune process. Based on this, ChatGPT suggested that gout was less likely and that immune arthritis was the primary explanation, possibly coexisting with a history of gout.

This conclusion represents a hallucination because it overemphasized one clinical feature (symmetry) while discounting several highly specific indicators of gout. The patient had a documented history of gout, elevated uric acid at the time of symptoms, nocturnal worsening of inflammation, and was already using colchicine—factors that strongly point toward a gout flare, even when the joint distribution is atypical. Gout can present symmetrically, especially in recurrent or polyarticular forms, and elbow involvement is well recognized in gout.

The hallucination did not arise from missing or invented facts. All patient details were correctly identified and restated. The error occurred because the reasoning gave disproportionate diagnostic weight to symmetry, treating it as a rule rather than a tendency. As a result, the model dismissed a coherent explanation—recurrent gout—that accounted for all reported symptoms, and instead favored an immune arthritis diagnosis that fit only part of the picture.

In summary, the hallucination lies in the misinterpretation and misweighting of clinical evidence. ChatGPT incorrectly concluded immune arthritis by prioritizing symmetry over the patient’s gout history, biochemical findings, symptom timing, and treatment response, leading to a plausible-sounding but incorrect diagnosis.

## 9. Related Work

### 9.1. Uncertainty, Surprise, and Information–Theoretic Approaches to Hallucination Detection

A prominent line of work in hallucination detection treats hallucinations as manifestations of uncertainty, surprise, or distributional shift. These approaches are rooted in inductive reasoning: they assume that hallucinated content deviates statistically from the training distribution or from the provided context, and can therefore be detected via entropy, likelihood, or information-theoretic measures. Recent work has leveraged token-level entropy, minimum log-probability, calibrated uncertainty, and information gain to flag potentially unreliable generations.

From a philosophical standpoint, these methods correspond to a narrow form of inductive inference. As emphasized in post-positivist philosophy of science [35,51,52], inductive inference is powerful for extrapolating observed regularities but fundamentally limited in its ability to justify novel explanatory content. Induction cannot, by itself, introduce new theoretical concepts or unobserved causal structures—precisely the kind of content that explanations require.

This limitation becomes particularly salient in the context of LLM hallucinations. A model may produce a low-entropy, statistically confident continuation that is nevertheless explanatorily invalid. Yao et al. [53] illustrate this phenomenon by framing hallucination as an adversarial process: they show that hallucination-inducing prompts yield entropy patterns that differ from benign prompts, and propose entropy-based thresholds for detection. While effective in adversarial settings, such approaches remain insensitive to whether a low-entropy explanation is *epistemically adequate* or merely narratively fluent.

Information-gain–based detectors improve on raw entropy by identifying unsupported additions relative to the source context. However, IG-only methods implicitly assume that great surprise correlates with incorrectness. As a result, they struggle with cases where a surprising claim is nevertheless explainable via a simple and well-established hypothesis. This gap motivates the integration of abductive reasoning, which explicitly distinguishes between *surprise that admits explanation* and *surprise that reflects reasoning failure*.

### 9.2. Faithfulness, Explanation Verification, and xAI Perspectives

A second major research direction focuses on faithfulness and explanation verification, particularly in explainable AI and LLM evaluation. These approaches ask whether a model’s explanation is supported by evidence, internally consistent, or aligned with retrieved facts. Techniques in this category include LLM-as-Judge evaluators, retrieval-augmented verification pipelines, consistency checks across multiple generations, and multi-stage verification frameworks such as Chain-of-Verification.

HaluCheck [54] exemplifies this trend by providing a visualization-oriented framework that aggregates multiple hallucination metrics and allows users to compare outputs across LLMs and evaluators. While valuable for exploratory analysis, such systems largely treat hallucination likelihood as an externally computed score, leaving the underlying notion of explanation adequacy implicit.

Philosophical analyses of explanation cast doubt on whether such surface-level verification is sufficient. Harman’s [30] notion of Inference to the Best Explanation (IBE) assumes that explanatory goodness correlates with truth, but this assumption has been extensively qualified. Lipton [31], Psillos [55], and Douven [56] argue that reasoners rarely have access to the full space of possible explanations. Consequently, what is actually performed is an Inference to the Best Available Explanation (IBAE), where “best” is evaluated only within the confines of what is currently conceivable.

Crucially, as Lipton and others emphasize, even the best available explanation may be epistemically unacceptable—particularly in domains characterized by novelty, sparse evidence, or weak methodological constraints. Historical examples make this clear: animistic explanations of natural phenomena were once the best available explanations, yet fail to meet modern standards of empirical adequacy, causal coherence, and predictive power. This observation generalizes directly to LLM explanations, which may be locally coherent and rhetorically compelling while remaining methodologically unsound.

Modern XAI research reflects similar concerns. Medianovskyi and Pietarinen [57] argue that the burden of evaluating explanations should not rest solely on human judgment, but should be shifted—at least in part—to the AI systems themselves, provided they are capable of genuinely abductive inference. Their critique highlights a fundamental limitation of inductive and attribution-based XAI methods, which often reduce explanations to saliency maps or causal attributions without offering criteria for their acceptance or rejection.

### 9.3. Abduction and Neuro-Symbolic Reasoning Frameworks

Abduction occupies a central position in both philosophy of science and artificial intelligence as the inferential mechanism responsible for hypothesis generation. In contrast to induction and deduction, abduction is conceptually creative: it introduces new hypotheses, theoretical entities, or causal structures that are not contained in the premises. As post-positivist philosophers have long emphasized, the justification of such hypotheses cannot be reduced to definitional analysis, but must proceed via ampliative inference [35,51,52].

In AI, this insight is formalized in ALP [27]. ALP systems implement a structured inference cycle consisting of abductive generation of hypotheses, deductive testing against background knowledge and constraints, and inductive or probabilistic evaluation. Importantly, this cycle operationalizes IBAE rather than idealized IBE: the system can only infer among hypotheses that are expressible within its symbolic vocabulary and computational resources.

Modern neuro-symbolic systems inherit the same epistemic constraints. As Bader and Hitzler [58] and d’Avila Garcez et al. [59] argue, even when neural components expand the hypothesis space by generating latent patterns or candidate explanations, the symbolic reasoning layer can only evaluate those hypotheses that fit within its representational schema. The resulting system balances expressive generativity against logical evaluability, mirroring the philosophical trade-offs inherent in IBAE.

Recent work explores this balance in various ways. Shi et al. [60] train LLMs to perform abductive reasoning using expert demonstrations, followed by retrieval-based validation of generated causes. Li et al. [61] and Quach et al. [62] address the problem of missing premises in multi-hop reasoning by optimizing intermediate steps via reinforcement learning.

At a more theoretical level, Pietarinen and Beni [63] connect abduction to active inference and the free-energy principle, grounding abductive reasoning in a broader cognitive–biological framework inspired by Peirce’s semiotics and pragmatism. Dubois et al. [64] examine abductive inference under conditions of missing priors, proposing information-theoretic and likelihood-based criteria when Bayesian priors are unavailable. Together, these works highlight both the promise and the difficulty of abductive reasoning under epistemic uncertainty.

Despite these advances, abduction-only systems remain vulnerable to overly convenient explanations: hypotheses that restore entailment but fail to meet broader standards of plausibility or robustness. Recent critiques of Chain-of-Thought reasoning [14] argue that abduction can address this weakness by enforcing global coherence, supporting defeasible revision, and enabling competition among explanations. The present work builds directly on this insight by integrating abduction with information gain, discourse structure, and counter-abduction, thereby operationalizing epistemic constraints that are implicit—but rarely formalized—in prior neuro-symbolic approaches.

## 10. Conclusions

By embedding abduction into the entropy-based framework, hallucination detection becomes a structured evaluation of *conditional justifiability*. This integration enables systems not only to identify unsupported content but also to differentiate between benign hypothesis formation, plausible inference, domain-appropriate generalization, and genuine error—bringing the combined model significantly closer to human standards of reasoning and explanation.

The discourse-aware abductive framework introduced in this work provides a principled foundation for constructing and verifying complex explanations generated by LLMs. By integrating abductive inference with rhetorical structure analysis, the approach enables systems to distinguish central, hypothesis-bearing content from peripheral or contextual material, thereby strengthening both explanatory precision and hallucination detection. The value of this integration is evident across multiple application domains.

Counter-abduction is thus a foundational component of hallucination-resistant neuro-symbolic reasoning. By positioning rival explanations as defeaters of LLM-generated CoTs, counter-abductive reasoning transforms narrative reasoning into a competitive, evidence-driven process grounded in logic and discourse structure. This provides a unified theoretical and computational basis for hallucination detection and correction across medical analysis, legal reasoning, scientific interpretation, and general-purpose CoT verification.

In medical narratives, weighting discourse nuclei over satellite descriptions allows the system to focus abductive diagnosis generation on patient-relevant complaints rather than tangential remarks, improving causal hypothesis extraction. In legal reasoning, the framework supports more transparent argument evaluation by giving precedence to claims occurring in the conclusion or main argument segments while attenuating the influence of background information. In scientific writing, it enhances the identification of robust causal explanations by prioritizing claims derived from results and discussion sections over speculative or forward-looking commentary. Finally, in LLM verification, discourse-aware abductive logic programming offers a structured mechanism for identifying hallucinations: statements originating in low-weight, peripheral text segments can be selectively discounted, while central claims undergo rigorous consistency checking.

Taken together, these applications demonstrate that combining abductive reasoning with discourse structure provides a versatile and effective method for improving reasoning fidelity, ensuring interpretability, and increasing trust in neuro-symbolic systems across diverse high-stakes domains.

Advantages:

- Increases interpretability: Abductive hypotheses are justified by discourse roles.
- Improves precision: Ignores peripheral text when generating explanations.
- Enables alignment with human reasoning: Since humans emphasize nuclei when forming explanations.
- Supports hallucination detection: Contradictions in nucleus-derived claims outweigh peripheral inconsistencies.

“Counter-abduction strength of confirmation metrics dialog” refers to a structured, interactive process where

1. Abductive reasoning proposes initial explanations for observed evidence.
2. Counter-abduction introduces competing explanations.
3. Confirmation metrics quantitatively assess how well the evidence supports each hypothesis.
4. Dialog facilitates the comparison and discussion of these assessments to arrive at the most plausible explanation.

Our framework is powerful in any scenario requiring rigorous evaluation of competing hypotheses, ensuring that conclusions are well-supported by evidence. It combines logical reasoning, probabilistic assessment, and collaborative discussion to navigate complex, uncertain situations effectively.

Our evaluation confirms that discourse structure and counter-abduction jointly improve both the logical soundness and perceived credibility of AI reasoning. D-ALP not only infers plausible explanations but also tests their robustness against rival interpretations, substantially reducing hallucinations. These combined results highlight the promise of discourse-aware abductive reasoning as a foundation for verifiable, trustworthy neuro-symbolic AI systems. In practical applications, the abductive hallucination discovery should work on top of white, gray, and black-box families of approaches (Wu et al. [65]) to be most efficient [66].

Using web search frequencies to approximate the probabilistic components of MDL effectively turns explanation evaluation into a form of fact checking Viavia large-scale web evidence. By grounding hypotheses and their supporting statements in empirical web co-occurrence statistics, the method implicitly verifies whether a proposed explanation aligns with widely attested facts, conventional causal relations, or commonly observed patterns. In this sense, the approach functions similarly to evidence retrieval pipelines—mapping a claim to the web and measuring how well it is supported—but does so in a model-agnostic and distribution-free way.

At the same time, this strategy is more versatile than standard fact-checking. Rather than requiring explicit evidence passages or structured knowledge bases, the method leverages the web’s implicit probability distribution: the relative frequency of statements serves as a proxy for how “complex,” “unexpected,” or “unsupported” an explanation is under MDL. This allows the system to score explanations even when no clean supporting document exists, and to detect misleading but superficially plausible reasoning by measuring its mismatch with broad linguistic and factual usage.

Thus, web-based probabilistic reconstruction provides a lightweight but powerful mechanism for explanation assessment—combining the grounding benefits of fact checking with the flexibility and generality of information–theoretic modeling.

The code is available at https://github.com/bgalitsky/halluc_in_health/tree/master/abduction (accessed on 27 January 2026). 

The datasets are:

1. Process control dataset where we identify hallucination in process control algorithms (https://github.com/bgalitsky/halluc_in_health/blob/master/data/process_control_hallucination_500.json (accessed on 27 January 2026)).
2. Patient complaint dataset where we find hallucinations in diagnosis-making (https://github.com/bgalitsky/halluc_in_health/blob/master/prolog/data/diseases_with_patient_complaints1000.xlsx (accessed on 27 January 2026)).

### 10.1. Limitations

While web-search frequencies provide a convenient and scalable proxy for estimating description lengths, this approach introduces several important limitations. First, search-engine hit counts are inherently noisy and unstable. They vary across time, region, device, and even repeated queries, reflecting index fluctuations, ranking algorithms, and undocumented heuristics rather than true corpus frequencies. As a result, the estimated probabilities *p*(*H*) and *p*(*O*|*H*) may exhibit high variance and occasional discontinuities.

Second, web frequencies are highly sensitive to query formulation. Small changes in phrasing, ordering, or stemming can produce large differences in result counts. Synonyms, paraphrases, and domain-specific terminology further complicate interpretation. Because hypotheses and observations rarely have a unique canonical linguistic form, model comparison may be biased by the particular string chosen to represent each proposition. This sensitivity undermines the reproducibility and robustness of the MDL estimates.

Third, the web contains significant topical, linguistic, and geographical biases. High-frequency content often reflects media cycles, SEO-optimized text, misinformation, and commercial duplication rather than underlying factual priors. Thus, common hypotheses may be “simple” in an information–theoretic sense only because they are culturally salient, newsworthy, or sensationalized—not because they genuinely have low description length in a formal model class. Conversely, accurate but specialized scientific hypotheses may receive artificially high code lengths due to their limited online footprint.

Finally, this method lacks principled handling of joint or conditional queries. Co-occurrence counts such as *f*(*H*,*O*) depend heavily on query operators (“AND”, quotation marks, proximity constraints), each interpreted differently across search platforms. Consequently, the derived conditional code lengths *L*(*O*|*H*) inherit semantic ambiguities from the search interface itself.

Taken together, these factors mean that web-based description lengths should be viewed as *heuristic approximations* rather than precise statistical quantities. They are most reliable when used for coarse-grained hypothesis ranking, and should ideally be complemented by more controlled corpora, domain-specific knowledge bases, or formal probabilistic models when higher fidelity is required.

### 10.2. Reproducibility and Practical Deployment

Reproducibility is a central design objective of the proposed framework, particularly given its intended use in safety-critical and high-stakes domains. To this end, the system is constructed as a modular neuro-symbolic pipeline, in which each component—discourse parsing, information gain computation, abductive reasoning, counter-abduction, and probabilistic grounding—can be executed, logged, and evaluated independently. This modularity enables controlled ablation, component-level validation, and straightforward replacement of individual modules without affecting the overall architecture.

In practical deployment, all symbolic reasoning steps (abductive hypothesis generation, constraint checking, and counter-abduction) are fully deterministic given fixed inputs, background knowledge, and hyperparameters. Non-determinism arises only from upstream neural components, such as discourse parsing or entailment scoring. To mitigate this variability, we fix model versions, decoding parameters, and random seeds, and cache intermediate outputs (e.g., EDU segmentation, discourse relations, entailment judgments) so that repeated runs yield identical symbolic reasoning traces.

Web-scale frequency estimates, used as a proxy for abductive description length, introduce an additional source of variability due to temporal drift and query sensitivity. To ensure reproducibility, we employ query canonicalization, frequency caching with explicit time stamps, and configurable smoothing parameters. In deployment settings where strict reproducibility is required, the web-based estimator can be replaced by a static snapshot, a curated corpus, or a domain-specific knowledge base without altering the abductive scoring logic.

Finally, the framework exposes all intermediate artifacts—EDU weights, hypothesis costs, defeated explanations, and final IG* scores—making reasoning decisions transparent and auditable. This level of traceability supports not only experimental reproducibility but also post hoc analysis and regulatory review in real-world applications.

## Figures and Tables

**Table 1:** Mapping between abductive criteria and information–theoretic interpretation.

Abductive Criterion	Information–Theoretic Interpretation
Simplicity	Low description length of the hypothesis
Explanatory adequacy	Low conditional entropy (*H*(*O*;*H*))
Coherence	High mutual information (*I*(*H*;*O*))
Plausibility	High prior probability (*P*(*H*))
Surprise reduction	High likelihood/low bit-cost of data given (*H*)

**Table 3:** Extending the features of ALP with discourse information.

Aspect	In Classical ALP	With Discourse-Aware ALP
Observation	A set of atomic facts or predicates.	Clauses extracted from *nucleus* discourse segments (main claims).
Abducibles	Candidate explanatory literals.	Hypotheses aligned with *satellite* segments, weighted by rhetorical relation (e.g., Evidence, Elaboration).
Explanatory Preference	Minimality or cost-based.	Weighted abductive preference: prioritize hypotheses supported by nucleus–satellite strength and coherence relations.
Conflict Resolution	Based on logical consistency.	Also guided by discourse coherence: conflicting explanations that preserve discourse flow are preferred.

**Table 4:** Hallucination detection F1 across datasets.

Dataset	Baseline ALP	ProbALP	IG-Only	Disk-Abduction	IG-Abduction	IG-Abduction + Counter-Abduction
TruthfulHalluc	0.63	0.66	0.71	0.72	**0.79**	**0.86**
MedHalluc	0.63	0.68	0.73	0.75	**0.83**	**0.88**
eSNLI_Halluc	0.60	0.68	0.70	0.72	**0.77**	**0.84**
HotPot-Halluc	0.65	0.64	0.69	0.72	**0.80**	**0.87**
**Average**	0.63	0.66	0.71	0.73	**0.80**	**0.86**

Bolded values indicate the best performance.

**Table 5:** Inference efficiency and pruning.

System	Avg. Time (s)	Search Space Reduction (%)
Baseline ALP	1.00	–
ProbALP	1.35	–
Disk-Abduction	0.88	−12%
IG-Abduction	0.82	−18%
IG-Abduction + Counter-Abduction	**0.79**	**−21%**

Bolded values indicate the best performance.

**Table 6:** Logical inconsistency (lower is better).

System	Defeated Hypotheses (%)
Baseline ALP	19
ProbALP	15
Disk-Abduction	13
IG-Abduction	**7**
IG-Abduction + Counter-Abduction	**6**

Bolded values indicate the best performance.

**Table 7:** Ablation of scoring components.

Variant	Δ Accuracy (%)	Δ Consistency (%)
Disk-Abduction	+7	+6
IG-Only	+8	+7
IG-Abduction	**+15**	**+12**

Bolded values indicate the best performance.

**Table 8:** Human interpretability ratings.

System	Clarity	Coherence	Trust
Baseline ALP	3.1 ± 0.2	2.9 ± 0.2	2.8 ± 0.2
ProbALP	3.3 ± 0.2	3.0 ± 0.2	3.0 ± 0.2
Disk-Abduction	4.0 ± 0.1	3.8 ± 0.1	3.9 ± 0.1
IG-Abduction	4.4 ± 0.1	4.3 ± 0.1	4.2 ± 0.1

**Table 9:** Trust calibration.

System	Trust Before	Trust After	Δ Trust
Baseline ALP	0.58	0.65	+0.07
ProbALP	0.57	0.67	+0.10
IG-Abduction + Counter-Abduction	0.55	**0.78**	**+0.23**

Bolded values indicate the best performance.

**Table 10:** Contribution of counter-abduction.

Dataset	IG-Abduction	IG-Abduction + Counter-Abduction
TruthfulHalluc	0.79	**0.86**
MedHalluc	0.83	**0.88**
eSNLI_Halluc	0.77	**0.84**
HotPot-Halluc	0.80	**0.87**
**Average**	0.80	**0.86**

Bolded values indicate the best performance.

**Table 11:** Contribution to human trust and error reduction.

Metric	IG-Abduction	IG-Abduction + Counter-Abduction
Hallucination F1	0.81	**0.87**
False Positive Rate	0.14	**0.09**
Human Trust	4.1	4.5

Bolded values indicate the best performance.

**Table 12:** Overall performance summary.

Aspect	IG-Abduction + Counter-Abduction	Δ Over Baseline
Logical Accuracy	0.86	+23%
Runtime Efficiency	0.79 s	−21%
Consistency Errors	6%	−68%
Human Clarity	4.7/5	+52%
Human Trust	4.5/5	+61%
Trust Calibration	+0.23	+0.16

**Table 13:** Comparison with SotA hallucination detectors.

Method	Claim F1	Expl. F1	Joint F1	F1: Easy-Wrong Subset (Section 8.5)
LLM-as-Judge (Self-check, [45]. claim only)	81.2	73.2	67.9	43.2
LLM-as-Judge (Snowballed hallucinations [48])	**82.0**	77.0	**70.0**	47.0
Chain-of-verification (CoVe, [46])	71.4	67.2	60.1	**52.6**
Retrieval + NLI, FactScore, [49]	65.1	53.6	49.8	45.7
Consistency-based verification, Truth-o-Meter [50]	67.5	61.0	55.2	42.9
IG only (ours)	34.1	32.9	33.3	29.2
IG + Abduction (ours)	68.9	62.1	56.4	50.2
IG + Abduction + Counter-Abduction (ours)	76.6	**79.3**	69.2	52.4

**Table 14:** Robustness of hallucination detection to degraded *L*(*H_c_*).

Variant	Avg. Hallucination F1
IG-Abduction (original)	0.80
+Noisy *L*(*H_c_*) (±50%)	0.79
+Quantized *L*(*H_c_*)	0.78
+30% corrupted *L*(*H_c_*)	0.77
IG-Only (no *L*(*H_c_*))	0.71

**Table 15:** False positive rate under degraded *L*(*H_c_*).

Variant	False Positive Rate
IG-Abduction (original)	0.14
+Noisy *L*(*H_c_*)	0.15
+Quantized *L*(*H_c_*)	0.16
+30% corrupted *L*(*H_c_*)	0.17
IG-Only	0.22

**Table 16:** Counter-abduction robustness under corrupted *L*(*H_c_*).

Variant	Hallucination F1
IG-Abduction + Counter-Abduction	0.86
+30% corrupted *L*(*H_c_*)	0.84
+Quantized *L*(*H_c_*)	0.83

**Figure A1:**
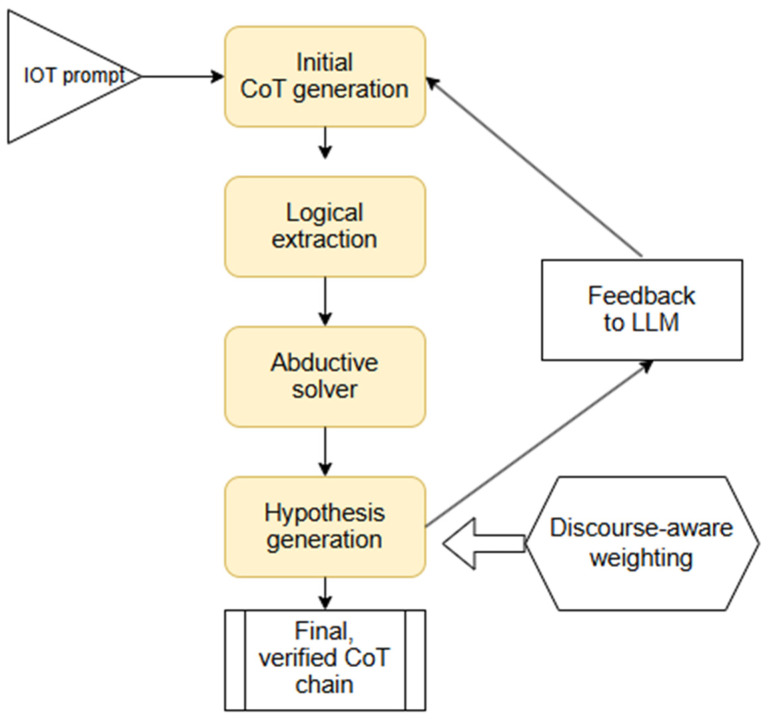
Abductive support for CoT.

## Data Availability

Data and code are available at https://github.com/bgalitsky/halluc_in_health/tree/master/abduction, accessed on 27 January 2026.

## Appendix A. Minimality as a Regularizer for CoT

Abductive models enforce minimality: explanations should contain no unnecessary assumptions. This principle acts as a structural regularizer on CoT, pruning verbose or extraneous content and discouraging speculative detours. Minimality also makes verification more tractable because the reasoning chain becomes closer to a canonical explanation.

Moreover, minimality reduces one of the main sources of hallucination in CoT systems: the inclusion of tangential premises or loosely associated facts. A minimal abductive explanation is not only easier to inspect but also more robust to adversarial perturbations and paraphrased prompts.

A coherent architecture for Abductive CoT emerges from combining the following elements (Figure A1):

1. Initial CoT generation by the LLM.
2. Logical extraction converting text into predicates or defeasible rules.
3. Abductive solver evaluates consistency, minimality, and coherence.
4. Hypothesis generation to fill explanatory gaps.
5. Feedback to LLM prompting revision or alternative reasoning paths.
6. Discourse-aware weighting using RST to distinguish central from peripheral content.
7. Final, verified CoT chain that satisfies explanatory constraints.

This loop is compatible with multiple logical formalisms, including probabilistic abduction, argumentation-based abduction, and paraconsistent abductive reasoning—allowing different degrees of uncertainty, conflict tolerance, and rule expressiveness. The core advantage is that the LLM no longer bears the full burden of reasoning; instead, it operates within a scaffold of symbolic constraints.

## Appendix B. Discourse-Weighted ALP (D-ALP)

Let *P* = (*Π*, *Δ*, *A*) be a standard abductive logic program:

- *Π*—strict rules;
- *Δ*—defeasible rules;
- *A*—set of abducibles.

We extend it with a discourse weighting function *w*:*l* → [0, 1] over literals L derived from RST trees:

- *w*(*l*) = 1.0 if *l* originates from a nucleus clause;
- 0 < *w*(*l*) < 1 if l originates from a satellite clause;
- *w*(*l*) = 0 if l appears in background or elaborative relations.

Then, the abductive explanation *E* ⊆ *A* minimizes:

$$Cost(E)\;=\;\sum_{l\in E}\left(1-w(l))\cdot penalty(l\right)$$

subject to *Π* ∪ *E* ⊨ *O*.

Thus, discourse prominence directly affects the search space and preference ordering among explanations.

The penalty function *penalty*(*l*) quantifies how “expensive” it is to *abduce* literal *l*—i.e., to assume *l* as true when it is not derivable from the strict rules *Π*. It represents epistemic risk: how far *l* departs from evidence, domain priors, or discourse plausibility.

*penalty*(*l*) = α⋅ρ(*l*) + β⋅κ(*l*) + γ⋅δ(*l*), where

- ρ(*l*) is a rule distance—number of rule applications needed to derive *l* (depth in derivation tree).
- κ(*l*) is a conflict measure—the degree to which *l* contradicts existing facts or competing hypotheses.
- δ(*l*) is a discourse mismatch measure—how incompatible *l* is with its rhetorical context (e.g., “Contrast”, “Condition”).

Constants α, β, γ control the importance of logical vs. discourse penalties (often α = 0.5, β = 0.3, γ = 0.2). Thus, penalty(*l*) is higher for:

- hypotheses that are logically remote;
- contradict evidence; or
- misalign with the discourse flow.

Suppose we have extracted the following from a clinical text (Table A1).

**Table A1:** characteristic of extraction from a clinical text.

Literal	Role	Rule Distance ρ	Conflict κ	Discourse Mismatch δ	Penalty(l) (Normalized)
disease(gout)	nucleus hypothesis	0.1	0	0.1	0.12
disease(arthritis)	competing hypothesis	0.1	0.4	0.2	0.22
disease(lupus)	irrelevant satellite	0.3	0.5	0.7	0.43

When we compute abductive cost with discourse weights:

- If w(gout) = 1.0 → cost ≈ 0;
- If w(arthritis) = 0.7 → cost ≈ 0.066;
- If w(lupus) = 0.4 → cost ≈ 0.26.

Hence, the D-ALP prefers the gout explanation: low penalty, high discourse weight.

## Appendix C. Discourse-Aware Abduction as Weighted Minimum Description Length

To integrate abductive reasoning with discourse-structured text, we model explanation selection as an optimization over hypotheses that best account for the discourse-segmented content of a model’s response. Let a response be decomposed into a sequence of Elementary Discourse Units (EDUs) ${\left\{{EDU}_{i}\right\}}_{i=1}^{n}$ using a RST–style parser. Each EDU is assigned a weight *w_i_* ≥ 0 reflecting its discourse salience, where *nucleus* units receive higher weights and *satellite* units receive lower weights, consistent with their respective rhetorical roles in encoding central vs. peripheral informational content.

For each EDU, we define *L*(${EDU}_{i}$|*H*) as the conditional description length of that EDU given *H*, interpreted as the residual amount of information needed to encode the EDU assuming the hypothesis is true. Formally, this may be instantiated as negative log-likelihood, information-theoretic coding length, or another monotonic cost metric encoding how well *H* renders the EDU unsurprising.

Discourse-aware abduction is then formalized as the following optimization problem:

(A1) $$H*=\;\underset{H}{\mathrm{arg min}}\left(L\left(H\right)+\sum_{i}w_{i}\;L\left({EDU}_{i}\;\right|H)\right)$$

This objective captures the dual desiderata of abductive inference:

1. parsimony of the hypothesis, enforced by *L*(*H*), and
2. explanatory adequacy relative to the discourse structure, enforced by the weighted sum of conditional description lengths.

The discourse weights *w_i_* ensure that explanatory pressure is concentrated on structurally central EDUs, while less critical satellite EDUs exert proportionally weaker influence. Equation (A1) therefore selects hypotheses that render the most important parts of the response informationally economical, reflecting the well-established RST assumption that nucleus content conveys the primary communicative intent.

Among all possible hypotheses H, choose the one that minimizes:

1. the complexity of the hypothesis itself;
2. the *discourse-weighted* cost of explaining each EDU.

It is an MDL objective, extended with discourse weights.

We are not just explaining “the text” as a whole; we are explicitly trying to explain each EDU.

Low *L*(*EDU_i_*|*H*) ≥ EDU is well explained by *H*;

High *L*(*EDU_i_*|*H*) ≥ EDU is surprising given *H*.

Explaining a central (nucleus) statement is more important than perfectly explaining every small detail (satellite). Each EDU is assigned a weight *w_i_* ≥ 0, derived from the discourse tree and taking into account specific discourse relations.

## Appendix D. EDU Example

To illustrate how discourse-aware abduction identifies medically implausible claims, consider a model-generated explanation segmented into four Elementary Discourse Units (EDUs) using an RST-style parser:

1. *EDU*_1_ (nucleus): “*The patient likely has gout.*”
2. *EDU*_2_ (nucleus; hallucinated): “*This gout is primarily caused by walking barefoot in cold seawater*.”
3. *EDU*_3_ (satellite): “*He has a history of elevated uric acid levels*.”
4. *EDU*_4_ (satellite): “*He frequently eats purine-rich foods such as red meat and seafood*.”

The RST analysis assigns *EDU*_1_ and *EDU*_2_ as nuclei, representing the core explanatory content, while *EDU*_3_ and *EDU*_4_ serve as satellites, providing contextual or supportive details. Because nuclei convey the primary communicative intent, they receive higher discourse weights, whereas satellites exert lower influence on explanatory selection. Let the weights be: *w*_1_ = 1.0, *w*_1_ = 0.8, *w*_3_ = 0.4, *w*_4_ = 0.3.

We evaluate the text under the discourse-aware MDL objective. To assess whether *EDU*_2_ can be explained or must be treated as a hallucination, we consider two competing hypotheses:

1. *H_med_*: Standard Medical Explanation “The patient has hyperuricemia and classical gout risk factors.” This hypothesis is clinically plausible and aligns with medical guidelines. Under *H_med_*, *EDU*_1_ (diagnosis of gout) is well explained; hyperuricemia is a canonical driver of gout → *low L*(*EDU*_4_|*H_med_*). *EDU*_3_ (history of elevated uric acid) fits directly → very low *L*. *EDU*_4_ (high-purine diet) is a well-known risk factor → low-to-moderate *L*.

*EDU*_2_, however, introduces a medically unsupported causal link between cold seawater and gout. Under any medically grounded hypothesis, this causal attribution is implausible → very high *L*. The hypothesis cost *L*(*H_med_*) is minimal because the hypothesis reflects standard medical reasoning.

2. *H_sea_*: Hallucination-Supporting Explanation. *H_sea_* = “Walking barefoot in cold seawater directly causes gout.” This is a non-standard and medically baseless causal theory. Under *H_sea_*, *EDU*_2_ (the hallucinated causal attribution) becomes fully explained → very low *L. EDU*_1_ (diagnosis of gout) becomes marginally more predictable → low *L*. *EDU*_3_ and *EDU*_4_ (uric acid history and diet) are poorly integrated into this hypothesis; they are neither predicted nor required → moderate-to-high *L*. Critically, the hypothesis itself is highly complex and unsupported by any medical evidence → very high *L*. Thus, explaining EDU_2_ under *H_sea_* incurs a large hypothesis penalty.

We now proceed to the evaluation of the discourse-weighted objective. For *H_med_*, there is a low hypothesis cost, a low residual for *EDU*_1_/*EDU*_3_/*EDU*_4_, but a high residual for *EDU*_2_. Weighted penalty is dominated by *w*_2_ *L*(*EDU*_2_|*H_med_*). For *H_sea_* there is an extremely high hypothesis cost L(*H_sea_*), reflecting the implausibility of the postulated causal mechanism. There are minor benefits from explaining *EDU*_2_: it does not compensate for the increased overall description length.

Since Score(*H_med_*) ≪ Score(*H_med_*) the system selects *H** = *H_med_*. No reasonable medical hypothesis can simultaneously remain simple (low *L*(*H*)) and make *EDU*_2_ unsurprising (low *L*(*EDU*_2_|*H*)). As a result, EDU_2_ receives a persistently high discourse-weighted cost.

Because *EDU*_2_ is a *nucleus*, its discourse weight is high (*w*_2_ = 0.8), amplifying the effect of its poor abductive fit. Even under the best hypothesis *H**, *L*(*EDU*_2_|*H**) remains large, and any attempt to reduce this cost (e.g., via *H_sea_*) inflates the hypothesis complexity term *L*(*H*) beyond acceptable bounds.

Thus, *EDU*_2_ is classified as: abductively unsupported, information-theoretically costly, and discourse-salient, and therefore constitutes a medical hallucination.

This extended example illustrates how discourse-aware abduction distinguishes between legitimate clinical extensions (*EDU*_1_, *EDU*_3_, *EDU*_4_) and unsupported causal inventions (*EDU*_2_), enabling a principled and interpretable mechanism for hallucination detection in medical reasoning.

This example is based on an actual hallucination produced by GPT-5.1, which incorrectly asserted that walking in cold seawater can precipitate a gout attack. The model generated a mechanistic but medically unfounded explanation by linking local cooling to urate crystallization, despite the absence of physiological evidence supporting such a causal mechanism. This illustrates how large language models can produce plausible-sounding but abductively unsupported medical claims, underscoring the need for discourse-aware, entropy-based hallucination detection.

## Appendix E. Counter-Abduction for Detecting Oversimplified Explanatory Hallucinations

A distinctive class of hallucinations addressed in this work concerns situations in which a model generates a claim that appears easily explainable from the given premises, yet the explanation it relies upon is incorrect or excessively superficial. In such cases, the claim itself may well be true, but the *inferential route* leading to it is flawed. This phenomenon arises when the model identifies a causally appealing but domain-inadequate explanatory shortcut—an abductive leap driven more by intuitive simplicity than by the underlying domain mechanisms.

Consider the common misconception that a gout attack can be caused by walking in cold water. On the surface, the abductive pathway is straightforward: *cold exposure* → *uric acid crystallization* → *gout flare*. This explanation is compact, causally intuitive, and readily generated by an LLM. However, it is medically incorrect. Gout flares depend primarily on systemic urate load, metabolic triggers, dietary factors, and local inflammatory processes; cold exposure may modulate symptoms but is not itself a causal trigger. Thus, while the event (“a gout flare occurred after walking in cold water”) may be true, the explanation is invalid precisely because it is *too easy* relative to the domain’s real causal structure.

Counter-abduction provides a principled mechanism for identifying such errors. Whereas standard abduction seeks the most plausible explanation consistent with the premises, counter-abduction introduces explicit *competition* among explanations. The system generates not only a candidate abductive explanation but also alternative counter-explanations that challenge its plausibility. These counter-abductions encode more accurate or more domain-coherent mechanisms for the same phenomenon and thereby serve as defeaters for oversimplified reasoning.

Operationally, counter-abduction proceeds in three steps. First, an abductive explanation is produced for why the claim might hold. Second, the system constructs counter-hypotheses that demonstrate either (a) how the same premises do *not* support the claim under correct causal interpretation, or (b) how the claim, if true, would more plausibly arise from mechanisms absent from the premises. Third, the abductive explanation is evaluated against these counter-hypotheses. If a counter-abduction offers a better, richer, or more medically grounded account, it *defeats* the original explanation, indicating that the model relied on an invalid or overly convenient reasoning path.

This defeat relation is central for hallucination detection. Unlike approaches that focus solely on factual contradictions or fabricated content, counter-abduction targets flawed explanatory structures. It allows us to flag answers in which the claim is not the problem—but the justification is. In safety-critical domains such as medicine or law, these explanation-level hallucinations are particularly dangerous, as they may persuade users with coherent yet incorrect causal narratives.

By requiring explanations to withstand competition from counter-explanations, counter-abduction mitigates the tendency of LLMs to prefer low-complexity, heuristically salient causal links. It ensures that abductive reasoning is not accepted merely because it looks plausible but only if it remains valid when confronted with alternative, domain-informed reasoning paths. In doing so, counter-abduction offers a structurally grounded approach for identifying and defeating “too-easy” explanations that underlie a subtle but important form of hallucination.
